# The synergistic effect of dielectric barrier discharge plasma and phycocyanin on shelf life of *Oncorhynchus mykiss rainbow* fillets

**DOI:** 10.1038/s41598-024-59904-9

**Published:** 2024-04-22

**Authors:** Maedehsadat Seyedalangi, Amir Hossein Sari, Bahareh Nowruzi, Seyed Amir Ali Anvar

**Affiliations:** 1 Department of Physics, Faculty of Converging Sciences and Technologies, Science and Research Branch, Islamic Azad University, Tehran, Iran; 2 Department of Biotechnology, Faculty of Converging Sciences and Technologies, Science and Research Branch, Islamic Azad University, Tehran, Iran; 3 Department of Food Hygiene, Science and Research Branch, Islamic Azad University, Tehran, Iran

**Keywords:** Dielectric barrier discharge plasma, Phycocyanin pigment, *Oncorhynchus mykiss rainbow* fillets, Shelf life, Biological techniques, Biotechnology, Microbiology

## Abstract

This study aimed to evaluate the efficacy of dielectric barrier discharge treatment (DBD) combined with phycocyanin pigment (PC) in extending the shelf life of *Oncorhynchus mykiss rainbow* fillets stored at 4 ± 0.1 °C. Microbiological, physicochemical, sensory and antioxidant properties were assessed over an 18-day storage period. The combined DBD and PC treatment significantly inhibited total viable counts and Psychrotrophic bacteria counts compared to the rest of the samples throughout storage. While Total Volatile Nitrogen concentrations remained below international standard until day 18, they exceeded this threshold in control sample by day 9. DBD treatment notably reduced Trimethylamine levels compared to controls (*p* < 0.05). PC and DBD combined inhibited DPPH and ABTS radical scavenging capacities by 80% and 85%, respectively, while demonstrating heightened iron-reducing antioxidant activity compared to controls. Analysis of 24 fatty acids indicated that PC mitigated DBD’s adverse effects, yielding superior outcomes compared to controls. The ratio of n-3 to n-6 fatty acids in all samples met or fell below international standard. Thus, the combined use of DBD and PC shows promise in extending fillet shelf life by over 15 days at 4 °C.

## Introduction

Nutrition plays a crucial role in determining human life quality, and one dietary trend on the rise is increased seafood consumption, driven by factors such as growing awareness of its associated health benefits. Seafood, recognized for its high protein content, omega-3 fatty acids, vitamins, and minerals, is often perceived as a healthier alternative to red meat due to its lower saturated fat content^1,2^. The combination of taste, nutritional value, sustainability, culinary adaptability, consistent availability, and alignment with health-conscious trends contributes to the significance of *Oncorhynchus mykiss* (Rainbow Trout) in the seafood industry^3^. Consumer satisfaction is heavily influenced by the key factor of freshness^3,4^.

Extending shelf life becomes imperative for consumer satisfaction, involving the inhibition of microbial growth, reduction of spoilage risks, and minimization of bacterial contamination. Utilizing modern methods like non-thermal plasma treatment and pigment incorporation, as opposed to traditional preservation approaches, offers substantial advantages in this regard^4,5^.

Non-thermal plasma, or cold plasma (CP), is a unique state characterized by partial ionization of gases at low temperatures. Energetic electrons induce this ionization, generating reactive oxygen species (ROS) and reactive nitrogen species (RNS). These species, derived from electron interactions with oxygen and nitrogen, partake in chemical reactions influencing both the plasma and surrounding surfaces. Also, CP is composed of various excited atomic, molecular, ionic, and radical species, coexisting with numerous reactive species such as electrons, positive and negative ions, free radicals, gas atoms, molecules in ground or excited states, and quanta of electromagnetic radiation (UV photons and visible light)^6^. CP finds diverse applications in medicine, materials science, and environmental engineering, showcasing potential benefits in wound healing, cancer treatment, sterilization, surface modification, and material functionalization^7^. Dielectric Barrier Plasma (DBD), characterized by a dielectric material between electrodes, allows controlled and localized plasma discharge. Studies, including Olatunde, et al.^8^ research, highlight the effectiveness of DBD plasma in increasing the lifespan of fish fillets^9^.

Stringent regulatory restrictions on synthetic colors and additives, prompted by potential health concerns, have led food manufacturers to explore natural alternatives^10^. Phycocyanin pigments (PC) derived from spirulina, a cyanobacterium renowned for diverse health benefits, are gaining attention as a safe and natural solution. This utilization aligns with regulatory requirements, emphasizing the industry's commitment to providing safe, high-quality, and naturally preserved food products^11^. According to Nowruzi et al.^5^, PC has been shown to extend the shelf life of rainbow trout.

Considering the absence of prior research on the combined effects of DBD plasma and PC treatment on rainbow trout, the innovative amalgamation of DBD plasma and PC treatment for *Oncorhynchus mykiss rainbow* fillets presents a pioneering approach to extending shelf life. By significantly prolonging shelf life, this innovation not only reduces economic losses for the industry but also ensures consumers access fresher and nutritionally rich rainbow trout fillets. The impact spans economic, environmental, and consumer satisfaction realms, marking a significant advancement in seafood preservation practices.

However, implementing DBD plasma and PC treatment in the fish industry faces challenges such as cost, scalability, and regulatory compliance, requiring strategic approach. Nevertheless, opportunities arise in enhanced shelf life, sustainability appeal, technological advancements, and market differentiation, emphasizing the need for collaborative industry adoption. Balancing challenges and opportunities are crucial for successful large-scale implementation, aligning with consumer demands and industry standards.

The objective of this study is to explore the synergistic effects of DBD treatment at two distinct voltages (70 and 80 kV) and exposure times (2 and 5 min), in the presence and absence of PC. The examination will cover microbial and chemical properties, sensory attributes, and antioxidant properties of Rainbow trout over an eighteen-day storage period at refrigeration temperature (4 °C).

## Material and methods

### Chemicals

In this study, all the chemicals were analytical grades purchased from Hi-Media, Merck, and Sigma.

### Spirulina cyanobacteria

The cultivation process of Spirulina cyanobacteria involved obtaining a strain from the Cyanobacteria culture collection of Azad University and purifying it. Subsequently, the purified samples were cultured in Zarrouk’s liquid culture medium within a growth chamber maintained at 28 °C ± 2. The cultivation spanned 30 days under continuous fluorescent light, with an intensity of 300 µE/m^2^s^12^.

### Extraction and purification of PC and additives

The PC was derived from spirulina microalgae cultivated in Zarrouk’s medium. Phycobiliproteins were separated using centrifugation, washed with phosphate buffer, and lyophilized. Extraction involved mixing freeze-dried biomass with sodium phosphate buffer, followed by freezing, thawing, and centrifugation. The PC content was collected, freeze-dried, and stored at − 20 °C^5^. To assess stability, citric acid preservative was added to the PC solution. Purity and concentration were analyzed using spectrophotometry^13^. The purity of PC was calculated using formula1^14^:

1 $$\mathrm{Purity \,of\, PC}=\frac{{A}_{620}}{{A}_{280}}$$

MIC was determined against *E. coli* and *Staphylococcus aureus*, both alone and in combination with citric acid, using the standard reference method^13^.

### Sample preparation and treatment

**Figure 1:**
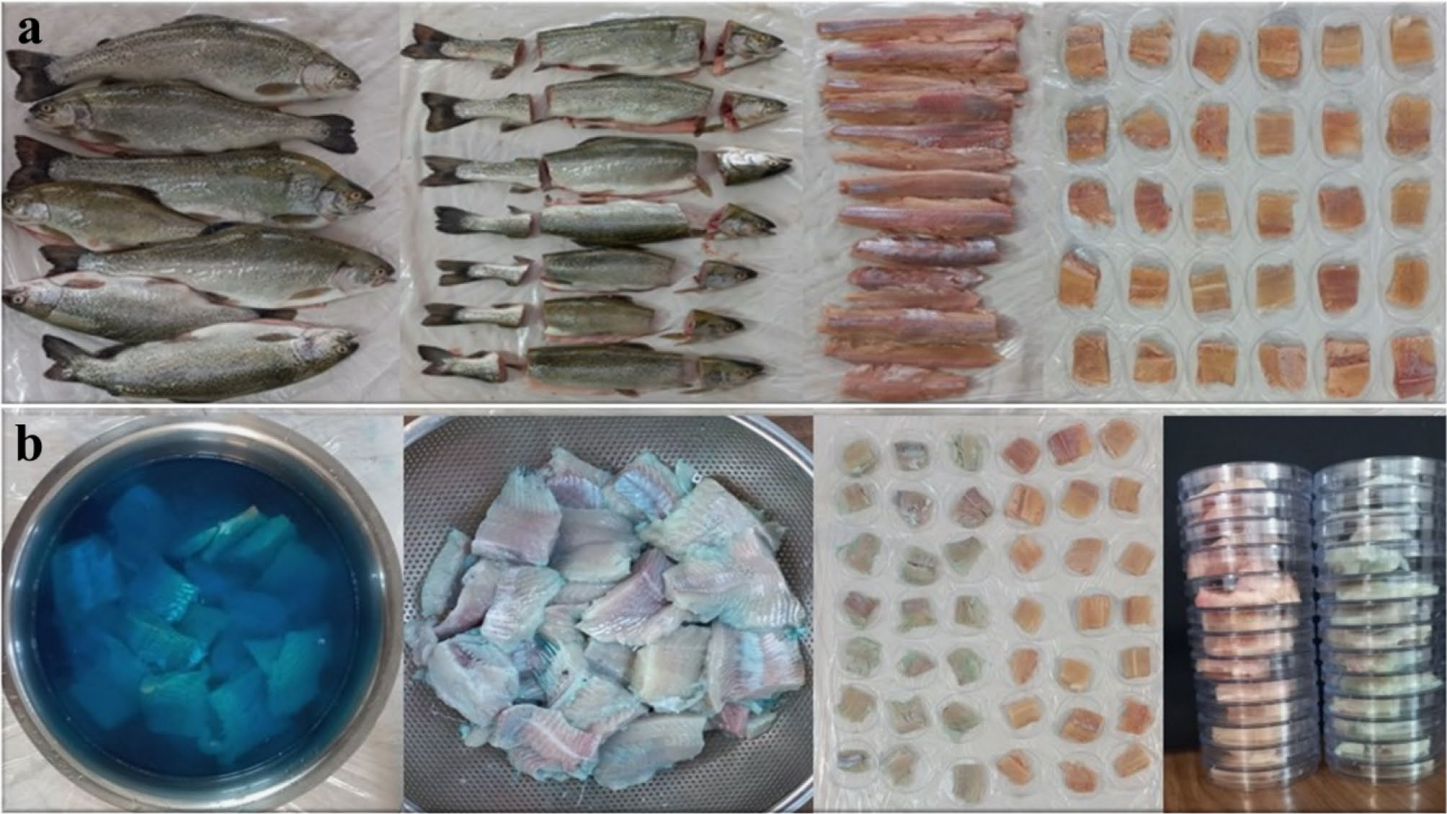
Sample preparation and treatment: (**a**) The process involves washing the fish, removing the head, tail, fins, and then filleting. (**b**) Fish fillets undergo treatment with PC pigment in preparation for plasma treatment.

*Oncorhynchus mykiss rainbow* fillets were obtained from the local market and processed in the laboratory. The fillets underwent trimming and were cut into standardized dimensions^15^ (5 cm in length, 5 cm in width, and 1 cm in thickness). Half of the fillets were pre-treated with PC, and all fillets were arranged in a Petri dish for DBD treatment at two different voltages (70 kV and 80 kV) and durations (2 min and 5 min) (Figs. 1a and 1b). A total of 200 fillets underwent ten different treatments. The sample abbreviations are as follows: Control sample without DBD and PC treatment: C, Sample treated with PC without DBD treatment: PC-P, Sample treated with DBD at 70 kV for 2 min and without PC treatment: P_70:2_−PC, Sample treated with DBD at 70 kV for 5 min and without PC treatment: P_70:5_−PC, Sample treated with DBD at 80 kV for 2 min and without PC treatment: P_80:2_−PC, Sample treated with DBD at 80 kV for 5 min and without PC treatment: P_80:5_−PC, Sample treated with combined treatment of PC and DBD at 70 kV for 2 min: P_70:2_+PC, Sample treated with combined treatment of PC and DBD at 70 kV for 5 min: P_70:5_+PC, Sample treated with combined treatment of PC and DBD at 80 kV for 2 min: P_80:2_+PC, Sample treated with combined treatment of PC and DBD at 80 kV for 5 min: P_80:5_+PC.

**Figure 2:**
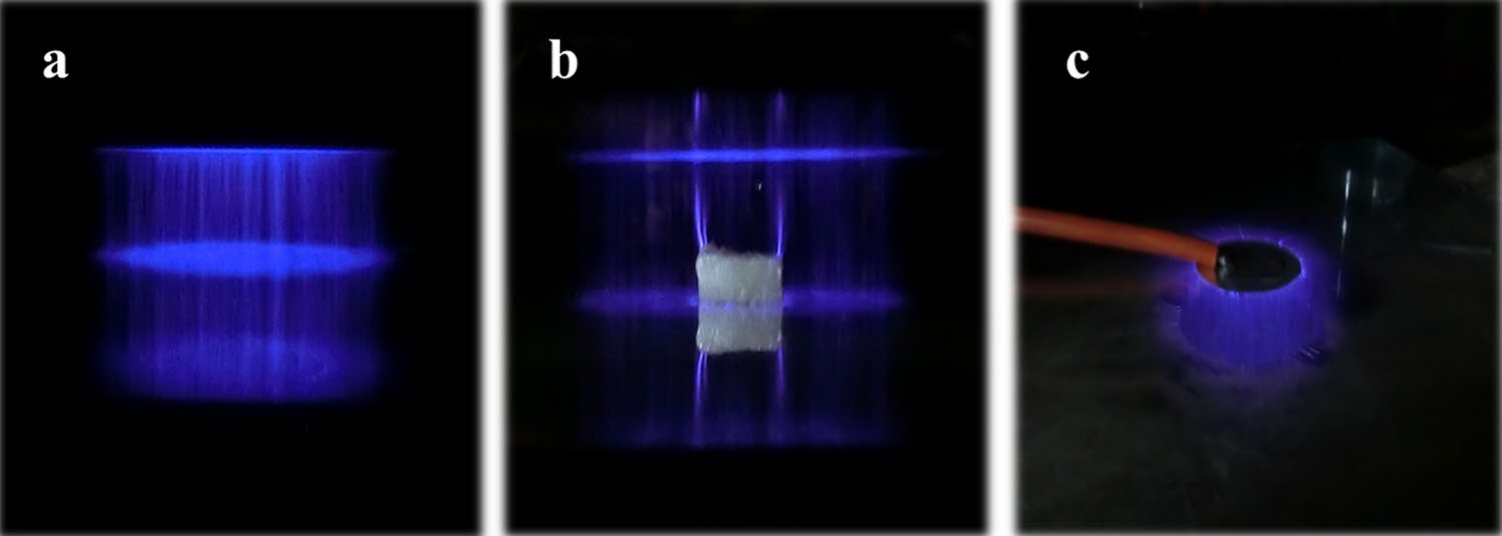
DBD Plasma Formation: (**a**) Plasma formation between two electrodes in side view, (**c**) Application of plasma treatment to fish fillets, and (**d**) Top view showing plasma formation.

The DBD system consists of two circular aluminum electrodes, each with a diameter of 15 cm, which were fabricated in the laboratory. One electrode is connected to a high-voltage (HV) power supply operating at 50 Hz, serving as the HV electrode, while the other is grounded. The electrodes are separated by a 3 mm thick glass dielectric with a fixed distance of 2 cm (Fig. 2). This distance remains constant for all experiments conducted. Following treatment, all fillets were stored at 4 °C and evaluated for microbiological, chemical, antioxidant, and sensory properties over an 18-day period at regular intervals (day 1, 3, 6, 9, 12, 15, and 18), with three replications for each evaluation.

To enhance treatment stability throughout the process, measures are implemented, including the use of standardized and sterilized Petri dish containers, precise sample arrangement, and quality control checks. Maintaining controlled laboratory conditions, such as consistent temperature and humidity, an optimal fixed distance between the plasma source and the sample, real-time monitoring and equipment calibration, along with conducting three replicates in all experiments, further contributes to treatment stability.

### Microbiological analyses

To accommodate the specific growth kinetics of the microorganisms under scrutiny, microbiological analyses were executed. Mean values from triplicate samples were recorded for all collected analysis results. Enumeration of total viable microorganisms (TVC) and psychrotrophic bacteria counts (TPC) was conducted using the spread plate technique^16^. Coagulase-positive *Staphylococcus aureus* bacteria (CoPS) were quantified utilizing Baird-Parker culture medium^17^. Enumeration of *Salmonella* and *Escherichia coli* bacteria was performed through the pour plate technique^18^. Lactobacillus bacteria counts (LB) were determined following established protocols^19^. The enumeration of Enterobacteriaceae (Ent) was carried out using an aerobic method^19,20^. Total counts were recorded and averaged, with data expressed as the logarithm of colony-forming units (CFU/g), and average and standard errors were calculated.

### Physicochemical properties

The samples underwent chemical analyses to determine their PH, titratable acidity^21^, peroxide number (PV)^22^, and thiobarbituric acid (TBA) values^23^. These analyses were performed in triplicate, and the mean value was recorded for each sample. The calculation of the Malondialdehyde (MDA) content in fillets, determined through Thiobarbituric Acid reactive substances )TBARS(, was conducted using the formula 2 and 3@@^24^:

2 $$c \left(\mu mol{L}^{-1}\right)=6.45 \left({A}_{532}-{A}_{600}\right)-0.56\times {A}_{450}$$

3 $$MDA\, concentration \left(\mu mol{Kg}^{-1} dry\, weight\right)=\frac{c\times {v}_{r}\times {v}_{e}}{{v}_{d}\times m\times 1000}$$

To determine the trimethylamine (TMA) and total volatile nitrogen content (TVN), a nitrogen distillation column was utilized^25^. Additionally,The fatty acid profile of the fillets was also analyzed for C, PC-P, P_80:5_, and P_80:5_+PC samples^26^.

### Color

The surface color of the fillets stored at 4 °C on the specified days was measured in triplicate. A portable Hunter lab Mini scan/EX device with illuminant D65 (Hunter Associates Laboratory; Reston, VA, USA) and 10 standard observers were utilized to measure the values related to L* (lightness), a* (redness/greenness), and b* (yellowness/blueness). The equipment was calibrated using white and black standards. The ΔE was calculated using the formula 4@@^27^:

4 $$\Delta E=\sqrt{{\left(\Delta {L}^{*}\right)}^{2}+{\left(\Delta {a}^{*}\right)}^{2}+{\left(\Delta {b}^{*}\right)}^{2}}$$

where $\Delta {{\text{L}}}^{*}$, $\Delta {{\text{a}}}^{*}$, and $\Delta {{\text{b}}}^{*}$ stand for the differences between the colors, which are characteristics of the samples and those colors of the control samples on the first day.

### Optical emission spectroscopy (OES)

A UV–Vis spectrometer, equipped with a fiber optic input, was used to obtain the emission spectrum of the discharge (Supplementary Fig. 1). Corresponding waveform and voltage-current diagrams were obtained at both voltages^19^.

### Antioxidant evaluation

The study focuses on analyzing the Diphenyl-1-Dipicrylhydrazyl (DPPH) assay in fish fillets. Minced fish fillet is mixed with an ethanol reagent and subsequently exposed to a 60 °C water bath for one hour. The resulting extract is then incubated with a DPPH solution in a dark room. The absorbance of the mixture is measured at a wavelength of 515 nm, and the final inhibition rate is calculated using the formula 5@@^24^.

5 $$DPPH\, \mathrm{scavenging\, rate }\left(\mathrm{\%}\right)= \frac{{\text{A}}0-A{\text{s}}}{{\text{A}}0}\times 100$$

Acording to the formula 5, A0 and As represent the absorbance values of the mixture prepared by 400 ml ethanol or 400 ml fillets extract mixed with 5.6 ml DPPH solution, respectively.

The antioxidant activity of fish fillets is assessed using the Ferric Reducing Antioxidant Power (FRAP) method. The FRAP assay is performed by incubating minced fish fillet with the FRAP solution for 30 min at 37 °C, followed by measuring the absorbance at 593 nm^28^.

To investigate the ABTS radicals assay in fish fillets, minced fish is added to the ABTS solution and incubated in a dark room. The range of color change is utilized as an indicator of antioxidant activity, and the percentage of inhibition of ABTS + (cationic radical) is calculated using the formula 6@@^29^:

6 $$\mathrm{inhibition \,rate }(\mathrm{\%})=\boldsymbol{ }\frac{\mathrm{Absorbance \,Control}-\mathrm{Absorbance \,sample}}{\mathrm{Absorbance\, control}} \times 100$$

### Sensory evaluation

The evaluation of fish fillets' quality is conducted through sensory testing. A panel of ten proficient assessors, comprising of five females and five males, evaluates the sensory attributes of the fillets, including color, odor, texture, and overall acceptability. This evaluation is performed using a 5-point hedonic scale ranging from 1 (extremely good) to 5 (extremely unacceptable). Prior to assessment, the fillet samples are heated in an oven bag at 180 °C for 10 min^30^.

### Statistical analysis

The study investigates the influence of various factors, such as voltage, time of DBD treatment, and addition of PC, on the shelf life of *Oncorhynchus mykiss rainbow* fillets. Microbiological, chemical, antioxidant, and sensory properties of the fillets are analyzed on days 1, 3, 6, 9, 12, 15, and 18, in triplicate, and compared with control fillets. statistical analysis is performed using SPSS software, employing One-way analysis of variance with 95% confidence intervals to identify significant differences among the variables. Means are compared using Tukey's test, and The results are presented graphically^31^.

### Ethics approval and consent to participate

All methods were carried out in accordance with relevant guidelines and regulations. Authors don’t do any experiments on humans and/or the use of human tissue samples. All experimental protocols and panelists involved in the study were approved by ethics committee of Tehran medical sciences, Islamic Azad University, Tehran, Iran. Moreover evaluating panel sampled the rainbow trout (Oncorhynchus mykiss) fillets. The full name of the ethics committee that approved the study is Dr Fahimeh Nemati and Dr Sarvenaz Falsafi.

## Results

### Purity, concentration and absorption spectrum of PC extracted from spirulina microalgae

The concentration and purity of PC was measured both before and after dialysis. Initially, the concentrations were found to be 0.238 mg/ml and 0.063 mg/ml, respectively. After dialysis, there was a slight increase in concentrations to 0.251 mg/ml and 0.065 mg/ml, indicating a minor degree of purification. The specificity of PC was determined by constructing a graph (Supplementary Fig. 2). The peak of this graph was found to be 621.9, indicating a high degree of specificity.

### Microbiological changes in *Oncorhynchus mykiss rainbow* fillets treated with DBD and PC during 18 days at 4°C

#### TVC and TPC

Notably, DBD treatment of P_80:5_ extended the shelf life of fish up to 9 days, while the combined effect of DBD treatment and PC in P_80:5_+PC the shelf life up to 12 days, indicating potential for the combination as a preservation method for fish products.

A significant increase in the TVC was observed across all samples during an 18-day storage period at 4 °C (*p* < 0.05) (Table 1). Notably, the C sample exhibited the highest increase in TVC, rising from 4.05 to 11.62 log CFU/g , while the P_80:5_+PC sample showed the lowest increase, rising from 3.52 log CFU/g to 9.66 log CFU/g (*p* < 0.05). Furthermore, on the third, sixth, ninth, and twelfth day of storage, the TVC exceeded the permissible limit of 6.00 log CFU/g for the C sample (6.39 log CFU/g ), PC-P (6.78 log CFU/g ), P_80:5_−PC (7.05 log CFU/g ), and P_80:5_+PC (6.69 log CFU/g ), respectively. Notably, DBD treatment of P_80:5_−PC extended the shelf life of fish up to 9 days, while the combined effect of DBD treatment and PC in P_80:5_+PC increased the shelf life up to 12 days.

**Table 1:** TVC changes with DBD treatment at 70 and 80 kV voltages and durations of 2 and 5 min, with and without PC treatment, on *Oncorhynchus mykiss rainbow* fillets during an 18-day storage period at 4°C.

TVC	Day1	Day3	Day6	Day9	Day12	Day15	Day18
C	4.05 ± 0.0047(a)(A)	6.39 ± 0.0041(a)(B)	7.00 ± 0.0140(a)(C)	8.31 ± 0.0055(a)(D)	8.91 ± 0.0108(a)(E)	10.90 ± 0.0266(a)(F)	11.62 ± 0.0256(a)(G)
P70:2−PC	3.96 ± 0.0088(a) (A)	5.97 ± 0.0165(b)(B)	6.39 ± 0.0042(b)(C)	7.37 ± 0.0090(b)(D)	8.38 ± 0.0068(b)(E)	10.35 ± 0.0099(b)(F)	11.17 ± 0.0052(b)(G)
P70:5−PC	3.84 ± 0.0197(bc) (A)	5.76 ± 0.0236(c)(B)	6.18 ± 0.0025(c)(C)	7.26 ± 0.0056(c)(D)	8.18 ± 0.0060(c)(E)	10.22 ± 0.0109(c)(F)	10.97 ± 0.0149(c)(G)
P80:2−PC	3.38 ± 0.0015(d) (A)	5.27 ± 0.0026(de)(B)	6.06 ± 0.0087(d)(C)	7.15 ± 0.0062(d)(D)	7.97 ± 0.0100(d)(E)	9.58 ± 0.0453(d)(F)	10.81 ± 0.0080(d)(G)
P80:5−PC	3.00 ± 0.0057(e)(A)	5.15 ± 0.0120(ef)(B)	5.67 ± 0.0111(e)(C)	7.05 ± 0.0138(e)(D)	7.35 ± 0.0060(e)(E)	9.36 ± 0.0038(e)(F)	10.29 ± 0.0058(e) (G)
PC-P	4.30 ± 0.0026(f)(A)	5.59 ± 0.0399(g)(B)	6.78 ± 0.0063(f)(C)	7.15 ± 0.0148(d)(D)	7.83 ± 0.0134(f)(E)	10.52 ± 0.0197(f)(F)	11.36 ± 0.0072(f)(G)
P70:2+PC	4.19 ± 0.0071(g)(A)	5.39 ± 0.0046(d)(B)	6.30 ± 0.0037(g)(C)	6.95 ± 0.0114(f)(D)	7.56 ± 0.0242(g)(E)	10.34 ± 0.0077(bc)(F)	11.33 ± 0.0102(f)(G)
P70:5+PC	3.95 ± 0.0200(ab)(A)	5.16 ± 0.0084(ef) (B)	5.88 ± 0.0068(h)(C)	6.69 ± 0.0177(g)(D)	7.26 ± 0.0076(h)(E)	10.03 ± 0.0081(g)(F)	11.08 ± 0.0073(g)(G)
P80:2+PC	3.79 ± 0.0157(c)(A)	5.07 ± 0.0129(f)(B)	5.55 ± 0.0390(i)(C)	6.10 ± 0.0090(h)(D)	7.03 ± 0.0085(i) (E)	9.31 ± 0.0109(e)(F)	10.24 ± 0.0124(e)(G)
P80:5+PC	3.52 ± 0.0594(h)(A)	4.48 ± 0.0611(h)(B)	5.40 ± 0.0020(j)(C)	5.56 ± 0.0139(i)(C)	6.69 ± 0.0191(j)(D)	8.67 ± 0.0384(h)(E)	9.66 ± 0.0142(h)(F)

Lowercase letters indicate significant changes in the column and uppercase letters indicate significant changes in the row.

A significant increase in the TPC was observed across all samples during an 18-day storage period at 4 °C (*p* < 0.05) (Supplementary Table 1). The C sample exhibited the highest increase in TPC, from 3.38 to 9.69 log CFU/g , while the P_80:5+_PC sample showed the lowest increase, from 2.53 to 7.55 log CFU/g (*p* < 0.05). DBD treatment effectively reduced TPC in all samples by increasing the voltage and the duration. However, the combined effect of DBD treatment with PC yielded more optimal results.

The rate of increase in TVC and TPC during the 18-day storage period in P_80:5_−PC and P_80:5_+PC samples was slower compared to the control sample. Moreover, the combined effect of DBD treatment with PC, especially in the case of P_80:5_+PC, inhibited the rate of increase in TVC and TPC significantly (*p* < 0.05).

#### Staph

There is a significant increase in Staph levels among all samples during the 18-day storage period at 4 °C (*p* < 0.05) (Supplementary Table 2). The C sample exhibited the highest increase in Staph rising from 0 to 9.33 log CFU/g. Conversely, P_80:5_+PC demonstrated the lowest increase in Staph levels, reaching only 7.09 log CFU/g. Moreover, on the 18th day, there is no significant difference observed between the C sample, P_80:2_−PC, P_70:5_−PC, and P_80:2_−PC (p > 0.05). Similarly, no significant difference was found on the 18th day between P_80:5_+PC, P_70:5_+PC, and P_80:5_+PC samples (*p* > 0.05). Interestingly, on the 18th day, the PC-P sample exhibited a significantly lower value of CPS (8.00 CFU/g ) than the C sample (p < 0).

#### *Salmonella* and *E. coli*

Throughout the 18-day storage period at 4 °C, neither the control nor the treated fish fillets showed any presence of *Salmonella* or *E. coli* bacteria. This finding underscores the effectiveness of the applied treatment in preserving the safety and quality of the fillets under refrigeration.

#### Lb

On the first day, the C sample contains 0.22 log CFU/g of LB. However, with DBD treatment (70 kV, 2 min), this value significantly decreased to 0.09 log CFU/g on the same day (*p* < 0.05) (Supplementary Table 3). As the voltage and treatment time increased, the Lb count in fish fillets decreased significantly, ultimately reaching zero (*p* < 0.05). Additionally, the presence of PC in DBD-treated fillets also resulted in a complete eradication of Lb.

The C sample exhibited the highest increase in Lb over 18 days, escalating from 0.22 to 9.39 log CFU/g (*p* < 0.05). However, PC alone effectively slowed down the growth of Lb. The lowest Lb count was observed on the 18th day, with a significant difference compared to the C sample observed in the P_80:5_+PC sample (7.88 log CFU/g).

Notably, on the 6th day, the Lb count in the control sample exceeded the spoilage limit of Lb (6 log CFU/g), reaching 6.36 log CFU/g. importantly, all treated samples surpassed this limit by the 12th day, except for the PC-P_80:5_ sample, which exceeded it on the 15th day, with a value of 6.84 log CFU/g (*p* < 0.05).

#### EB

The initial number of EB in all samples is zero on the first day (Supplementary Table 4). The highest and lowest levels of EB are observed on the 18th day of storage for the C sample (9.83 log CFU/g) and P_80:5_+PC sample (6.85 log CFU/g), respectively. The C sample exhibits the greatest increase in EB during the 18-day storage period, with levels rising from 0.00 to 9.83 log CFU/g (*p* < 0.05). DBD treatment, both with and without PC, leads to a decrease in the number of EB in all samples over time, with higher voltage and longer treatment time resulting in more significant reductions. Notably, DBD treatment with and without PC yields more favorable results on all days in all samples. Throughout the storage period, the P_80:5_+PC sample consistently demonstrates the lowest number of EB compared to the C sample, with a significant difference observed on the 18th day (6.85 log CFU/g).

### Physicochemical changes in *Oncorhynchus mykiss rainbow* fillets treated with DBD and PC during 18 days at 4 °C

#### PH and acidity

The PH values increase significantly in all samples during the 18-days storage period (Supplementary Table 5). The C sample exhibited the highest increase, from 6.33 on the first day to 7.73 on the 18th day (*p* < 0.05). On the first day, the pH value of the PC-P sample significantly increased compared to the C sample, from 6.33 to 6.44 (*p* < 0.05). However, except on the first day, the pH value of the PC-P sample is consistently lower than of both the C sample and samples treated only with DBD (*p* < 0.05). The inhibitory effect of PC on PH is more potent than DBD treatment alone. Throughout the 18-day storage period, the C sample exhibited the highest pH value, while the lowest pH value is observed in the P_80:5_+PC sample (*p* < 0.05).

The pH values decrease significantly in all samples treated with DBD, with or without PC, over the 18-day storage period. However, the pH changes are slower in samples treated with both DBD and PC compared to those treated with DBD alone and the C sample. The combined effect of DBD treatment with PC in inhibiting pH is stranger than the independent effect of each treatment. Furthermore, increasing the treatment time at a constant voltage of 80 kV causes a significant decrease in the pH value in all samples (*p* < 0.05). However, this reduction rate is not significant on the third day for P_70:2_−PC and P_70:5_−PC samples and on the first and sixth days for samples P_70:2_+PC, P_70:5_+PC, P_80:2_+PC, and P_80:5_+PC at a constant voltage of 70 kV (*p* > 0.05). Increasing the voltage at a fixed time of 5 min causes a significant decrease in the pH value in all samples (*p* < 0.05). However, this reduction rate is not significant at a constant time of 2 min on the 9th and 12th days for samples P_70:2_−PC, P_70:5_−PC, P_80:2_−PC, and P_80:5_−PC, and on the first and sixth days for samples P_70:2_+PC, P_70:5_+PC, P_80:2_+PC, and P_80:5_+PC (p > 0.05).

The acidity of all samples increased significantly over the 18-day storage period (*p* < 0.05) (Supplementary Table 6). The C sample exhibited the highest rate of acidity increase, reaching from 0.13 to 0.24, whereas the P_70:5_+PC and P_80:5_+PC samples showed the lowest rate of increase, reaching from 0.13 to 0.19 (*p* < 0.05). PC-P sample consistently exhibits lower acidity levels than the C sample throughout the entire 18-day period (p < 0.05), indicating the effective ability of PC to reduce acidity levels. Furthermore, the C sample consistently exhibits the highest levels of acidity throughout the entire storage period, with only the P_70:2_−PC sample showing no statistically significant difference on the 15th day (*p* > 0.05). In contrast, the P_80:5_+PC sample consistently exhibited the lowest levels of acidity, with no statistically significant difference from the P_70:5_+PC, P_70:5_−PC, and P_80:5_−PC samples except on the third day (*p* > 0.05). Samples treated solely with DBD showed lower acidity levels than the C sample, with no statistically significant difference from the PC-P sample on the first, third, 12th, 15th, and 18th days (*p* > 0.05). Additionally, combined treatment with DBD and PC significantly slowed down the rate of acidity increase compared to the control sample over the 18-day period (*p* < 0.05).

#### PV and TBARS

The PV of all samples increases significantly during the 18-day storage period (Supplementary Table 7). On the 18th day, the C sample exhibits the highest PV (0.55), while the lowest PV is observed in the P_80:5_+PC sample (0.38) (*p* < 0.05). Except for the first day, the PV of the PC-P sample is significantly lower than that of the C sample and the P_70:2_−PC sample (*p* < 0.05). The rate of PV increase during the 18-day storage period is slower in samples treated with DBD than in the C sample, and it is even slower in samples treated with combined DBD and PC than in samples treated with DBD alone and the C sample.

On the first day, no statistically significant difference is observed between the C sample and the samples treated only with DBD and the PC-P sample (p > 0.05). However, the PV of the P_70:2_+PC, P_70:5_+PC, P_80:5_+PC, and P_80:2_+PC samples was significantly lower than that of the C sample (*p* < 0.05). On the 18th day, there is no statistically significant difference in PV between the PC-P and P_80:2_−PC samples (*p* > 0.05). Except for the first day, increasing the treatment time (from 2 to 5 min) at a constant voltage of 80 kV causes a significant decrease in PV (*p* < 0.05). On the third day, this reduction rate in the constant voltage of 50 kV for the P_70:2_−PC and P_70:5_−PC samples do not create a significant statistical difference (*p* > 0.05). Additionally, increasing the voltage causes a significant decrease in PV on all days except the first day (*p* < 0.05). On the first day, there is no statistically significant difference between the C sample and the treated samples (*p* > 0.05).

TBARS levels increase in all samples during the 18-days of storage period, and this increase is significant (Table 2). However, the rate of increase is much faster in the C sample (0.21–0.97) compared to the treated samples, and this difference is statistically significant (*p* < 0.05). The initial values of TBARS in fillets are consistent with those reported for high-quality fish products (1–2 mg MDA/Kg). Notably, on the first day, DBD treatment does not result in any significant difference (*p* > 0.05). on the subsequent days, DBD treatment, with and without PC, resulted in a significant decrease in TBARS values compared to the C sample (*p* < 0.05). This decreases is more pronounced in the samples treated with a combination of DBD and PC. By the end of the 18-days of storage period, the TBARS values decreased from 0.97 mg MDA/Kg (C sample) to 0.69 mg MDA/Kg (P_80:5_−PC) and then to 0.60 mg MDA/Kg (P_80:5_+PC) (*p* < 0.05). Moreover, DBD treatment itself significantly reduces TBARS values during storage. Nonetheless, the combined treatment of DBD and PC is more effective in reducing TBARS values compared to each treatment independently.

**Table 2:** TBARS changes with DBD treatment at 70 and 80 kV voltages and durations of 2 and 5 min, with and without PC treatment, on *Oncorhynchus mykiss rainbow* fillets during an 18-day storage period at 4°C.

TBARS	Day1	Day3	Day6	Day9	Day12	Day15	Day18
C	0.21 ± 0.0019 (ad)(A)	0.36 ± 0.0030(a)(B)	0.44 ± 0.0069(a)(C)	0.62 ± 0.0040(a)(D)	0.75 ± 0.0019(a)(E)	0.86 ± 0.0019(a)(F)	0.97 ± 0.0030(a)(G)
P70:2−PC	0.19 ± 0.0030 (b) (A)	0.29 ± 0.0022(bd)(B)	0.40 ± 0.0034(b) (C)	0.60 ± 0.0022(b) (D)	0.68 ± 0.0019(b) (E)	0.81 ± 0.0039(b)(F)	0.87 ± 0.0030(b) (G)
P70:5−PC	0.20 ± 0.0052 (ac) (A)	0.25 ± 0.0030(c) (B)	0.38 ± 0.0040(c) (C)	0.55 ± 0.0040(c) (D)	0.65 ± 0.0019(c) (E)	0.69 ± 0.0019(c)(F)	0.78 ± 0.0019(c) (G)
P80:2−PC	0.19 ± 0.0019 (bc) (A)	0.24 ± 0.0081(c) (B)	0.35 ± 0.00040(df) (C)	0.51 ± 0.0039(d) (D)	0.63 ± 0.0040(d) (E)	0.70 ± 0.0030(c) (F)	0.79 ± 0.0040(ce) (G)
P80:5−PC	0.20 ± 0.0011 (bc) (A)	0.22 ± 0.0040(d) (B)	0.31 ± 0.0019(e)(C)	0.44 ± 0.0019(e) (D)	0.53 ± 0.0022(e) (E)	0.62 ± 0.0052(d)(F)	0.69 ± 0.0039(d) (G)
PC-P	0.23 ± 0.0022 (d) (A)	0.30 ± 0.0011(b)(B)	0.41 ± 0.0000(b) (C)	0.55 ± 0.0000(c) (D)	0.64 ± 0.0019(c) (E)	0.73 ± 0.0030(e) (F)	0.80 ± 0.0030(e) (G)
P70:2+PC	0.23 ± 0.0011 (d) (A)	0.28 ± 0.0000(ef) (B)	0.36 ± 0.0040(cd) (C)	0.43 ± 0.0000(e) (D)	0.53 ± 0.0034(e) (E)	0.60 ± 0.0049(f) (F)	0.67 ± 0.0019(f) (G)
P70:5+PC	0.23 ± 0.0022 (d) (A)	0.27 ± 0.0019(f) (B)	0.34 ± 0.0034(f) (C)	0.39 ± 0.0030(f) (D)	0.47 ± 0.0000(f) (E)	0.56 ± 0.0081(g) (F)	0.65 ± 0.0011(g) (G)
P80:2+PC	0.23 ± 0.0011 (d) (A)	0.25 ± 0.0019(c) (B)	0.31 ± 0.0040(e) (C)	0.36 ± 0.0019(g) (D)	0.46 ± 0.0030(g) (E)	0.54 ± 0.0022(h) (F)	0.62 ± 0.0022(h) (G)
P80:5+PC	0.23 ± 0.0030 (d) (A)	0.21 ± 0.0019(d) (B)	0.28 ± 0.0019(g) (A)	0.33 ± 0.0039(h) (C)	0.43 ± 0.0019(h) (D)	0.51 ± 0.0019(i) (E)	0.60 ± 0.0030(i)(F)

Lowercase letters indicate significant changes in the column and uppercase letters indicate significant changes in the row.

Importantly, on the 18th day, the lowest TBARS value with significant differences compared to the C sample is observed in the P_80:5_+PC sample (*p* < 0.05). It is noteworthy that a TBARS value higher than 2.0 mg MDA/Kg in fish indicates spoilage. However, after 18 days of storage, this value is less than 1 in all samples, and very close to 1 in the C sample. Furthermore, increasing the treatment time at a constant voltage of 80 kV results in a significant decrease in TBARS values on all days except the first day (*p* < 0.05). However, the reduction rate at the constant voltage of 70 kV on the third day for P_70:2_+PC and P_70:5_+PC samples do not create a significant statistical difference (*p* > 0.05). Additionally, increasing the voltage always results in a significant decrease in TBARS values on all days except the first day (*p* < 0.05). Notably, on the first day, there is no statistically significant difference between the C sample and the treated samples (*p* > 0.05).

#### TMA and TVN

The results show a significant increase in TVN across all samples during the 18-day storage period (*p* < 0.05) (Table 3). The C sample demonstrates a faster increase compared to treated samples, nearing the permissible limit by the ninth day, while the PC-P and P_80:5_−PC samples exceed the limit on the twelfth and fifteenth days, respectively. However, the P_80:5_+PC sample remains below the limit by the 18th day. Initially, all samples exhibit TVN values below 10 mgN/100 g, indicating good quality with no significant difference observed (*p* > 0.05). By the third day, all samples surpass the 10 mgN/100 g limit, except for the P_80:5_+PC sample, which maintains freshness until the sixth day. DBD treatment, with or without PC, reduces TVN values compared to the C sample on the 9th, 12th, 15th, and 18th days (*p* < 0.05). The PC-P sample consistently shows lower TVN values than the C sample, while combined treatments exhibit even lower TVN values, suggesting effective mitigation of TVN in treated samples, particularly with DBD and PC combinations.

**Table 3:** TVN changes with DBD treatment at 70 and 80 kV voltages and durations of 2 and 5 min, with and without PC treatment, on *Oncorhynchus mykiss rainbow* fillets during an 18-day storage period at 4 °C.

TVN	Day1	Day3	Day6	Day9	Day12	Day15	Day18
C	7.84 ± 0.3233(ab)(A)	17.55 ± 0.4938(a)(B)	24.92 ± 0.1616(a)(C)	34.63 ± 0.1866(a)(D)	41.53 ± 0.1866(a)(E)	47.60 ± 0.1616(a)(F)	53.76 ± 0.3233(a)(G)
P70:2−PC	7.84 ± 0.3233(ab) (A)	15.68 ± 0.3233(b)(B)	22.12 ± 0.1616(b) (C)	31.08 ± 0.3233(b) (D)	36.96 ± 0.1616(b) (E)	42.65 ± 0.2469(b)(F)	48.44 ± 0.1616(b) (G)
P70:5−PC	8.21 ± 0.1866(ab) (A)	15.12 ± 0.3233(bc) (B)	19.88 ± 0.1616(cd) (C)	28.84 ± 0.1616(c) (D)	34.25 ± 0.1866(c) (E)	38.36 ± 0.3233(c)(F)	43.87 ± 0.3733(c) (G)
P80:2−PC	7.84 ± 0.3233(ab) (A)	13.81 ± 0.1866(cd) (B)	18.85 ± 0.2469(ce) (C)	26.97 ± 0.2469(d) (D)	34.07 ± 0.2469(c) (E)	39.57 ± 0.0933(d) (F)	45.36 ± 0.0000(d) (G)
P80:5−PC	7.47 ± 0.1866(ab) (A)	11.95 ± 0.4938(e) (B)	15.68 ± 0.1616(f)(C)	24.64 ± 0.1616(e) (D)	30.71 ± 0.2469(d) (E)	35.75 ± 0.1866(e)(F)	41.25 ± 0.1866(e) (G)
PC-P	8.40 ± 0.3233(b) (A)	14.84 ± 0.1616(bc)(B)	22.40 ± 0.1616(b) (C)	31.08 ± 0.1616(b) (D)	37.24 ± 0.1616(b) (E)	43.77 ± 0.1866(f) (F)	49.00 ± 0.1616(b) (G)
P70:2+PC	8.21 ± 0.1866(ab) (A)	12.97 ± 0.2469(de) (B)	20.25 ± 0.1866(d) (C)	24.83 ± 0.0933(e) (D)	30.52 ± 0.1616(d) (E)	37.71 ± 0.0933(c) (F)	41.81 ± 0.2469(e) (G)
P70:5+PC	8.77 ± 0.3733(b) (A)	12.41 ± 0.0933(de) (B)	18.39 ± 0.1866(e) (C)	21.56 ± 0.1616(f) (D)	27.81 ± 0.1866(e) (E)	35.09 ± 0.3365(e) (F)	39.67 ± 0.1866(f) (G)
P80:2+PC	7.47 ± 0.1866(ab) (A)	11.20 ± 0.6466(e) (B)	15.96 ± 0.2333(f) (C)	19.60 ± 0.1616(g) (D)	25.48 ± 0.1616(f) (E)	32.48 ± 0.1616(g) (F)	37.05 ± 0.1866(g) (G)
P80:5+PC	6.91 ± 0.1866(a) (A)	8.77 ± 0.1866(f) (B)	13.63 ± 0.3365(g) (C)	16.80 ± 0.1616(h) (D)	22.68 ± 0.1616(g) (E)	29.21 ± 0.2469(h) (F)	33.69 ± 0.4938(h)(G)

Lowercase letters indicate significant changes in the column and uppercase letters indicate significant changes in the row.

The results show a significant increase in TMA values across all samples during the 18-day storage period, with the C sample exhibiting a notably faster increase than treated samples (Supplementary Table 8). Initially, no significant difference in TMA values is observed between treated samples and C (*p* > 0.05). By the 9th day, the C sample reaches 0.25 mg N/100g TMA, while the P_80:5_+PC sample remains below this value. By the 18th day, the C sample records the highest TMA value (53.76 mg N/100 g), whereas the lowest is observed in the P_80:5_+PC sample (33.69 mg N/100 g). Increasing treatment time at 80 kV voltage leads to a significant decrease in TMA values, more pronounced with longer treatment durations. However, this reduction is not significant for samples treated at 70 kV voltage on the ninth and twelfth days. Additionally, increasing voltage consistently decreases TMA values. Furthermore, PC, with or without DBD, significantly reduces TMA values compared to the C sample on all days except the first, indicating potential for TMA control in fish product storage with DBD and PC treatments.

### FFA in *Oncorhynchus mykiss rainbow* fillets treated with DBD and PC during 18 days at 4 °C

The FFA composition of the C, PC-P, P_80:5_−PC, and P_80:5_+PC samples is analyzed on the first day. 24 types of FFA have been identified, divided into three groups: saturated fatty acids (SFA), monounsaturated fatty acids (MUFA), and polyunsaturated fatty acids (PUFA) (Table 4).

**Table 4:** FFA values for four samples (C, PC, P_80:5 _− PC and P_80:5_+PC) in *Oncorhynchus mykiss rainbow* fillets at 4 °C.

Fatty acid	Name	C	PC-P	P_80:5_−PC	P_80:5_+PC
C14:0	Myristic acid	0.81^a^	0.79^b^	1.12^c^	0.85^d^
C15:0	Pentadecanoic acid	0.21^a^	0.20^b^	0.19^c^	0.19^a^
C16:0	Palmitic acid	11.67^a^	12.48^b^	15.80^c^	11.61^d^
C17:0	Heptadecanoic acid	0.21^a^	0.27^b^	0.23^c^	0.21^a^
C18:0	Stearic acid	4.64^a^	4.11^b^	4.56^c^	3.29^d^
C20:0	Arachidic acid	0.44^a^	0.34^b^	0.51^c^	0.51^c^
Saturated fatty acids^1^		16.1	18.19	22.41	16.66
C16:1	Palmitoleic acid	3.17^a^	3.00^b^	3.95^c^	3.15^d^
C17:1		0.16^a^	0.12^b^	0.22^c^	0.16^a^
C18:1 n9c	Oleic acid	35.36^a^	34.05^b^	30.37^c^	36.68^d^
C20:1	Gadoleic acid	0.19^a^	0.17^b^	0.22^c^	0.21^d^
Monounsaturated fatty Acids^2^		38.88	37.34	34.76	40.20
C18:2 n6c	LNA	32.34^a^	33.78^b^	32.00^c^	32.40^d^
C18:3 n6	γ-linolenic acid	1.17^a^	1.13^b^	1.22^c^	1.17^a^
C20:3 n6	Dihomo-γ-linolenic Acid	1.31^ac^	1.31^a^	0.96^b^	1.33^c^
C20:4 n6	ARA	0.04^a^	0.05^b^	0.04^a^	0.06^c^
C22:4 n6	DTA	0.20^a^	0.16^b^	0.15^c^	0.22^d^
C22:5 n6	Dpan-6	0.34^a^	0.35^a^	0.35^b^	0.32^c^
Omega 6 fatty acids^4^		35.40	36.78	34.72	35.50
C18:3 n3	ALA	2.02^a^	1.90^b^	2.81^c^	1.98^d^
C20:3 n3	Eicosatrienoic acid	0.96^a^	1.06^b^	0.64^c^	0.97^a^
C20:4 n3	Eicosatetraenoic acid	0.18^a^	0.23^b^	0.30^c^	0.26^d^
C20:5 n3	EPA	0.19^a^	0.27^b^	0.20^c^	0.20^c^
C22:5 n3	DPA	0.14^a^	0.14^a^	0.19^b^	0.13^c^
C22:6 n3	DHA	1.72^a^	1.48^b^	2.04^c^	1.58^d^
Omega 3 fatty acids^3^		5.21	5.08	6.18	8.07
C20:2	eicodienoic acid	1.17^a^	1.11^b^	0.86^c^	1.17^b^
C20:3 n9	mead acid	1.38^a^	1.51^b^	1.22^c^	1.41^d^
n-3/n-6ratio^5^		0.14	0.13	0.17	0.22

Lowercase letters indicate significant changes in the row.

#### SFA

The values of all SFAs decrease significantly compared to the C sample when PC is added to the samples, except for C16:0 and C17:0. Conversely, these values increase significantly in all samples compared to the C sample with DBD treatment, except for C15:0 and C18:0. However, the combined effect of PC and DBD treatment reduces the SFA values of all samples significantly compared to samples treated only with DBD. It is noteworthy that across all four samples, C16:0 and C18:0 exhibit the highest values, while C15:0 has the lowest value. In terms of total SFAs, P_80:5_−PC, PC-P, P_80:5_+PC, and C rank from highest to lowest, respectively.

#### MUFA

The values of all MUFAs decrease significantly compared to the C sample when PC is added to the samples. Conversely, these values increase significantly in all samples compared to the C sample with DBD treatment, except for the C18:1 n9c sample. However, the combined effect of PC and DBD treatment reduces the MUFA values of all samples significantly compared to samples treated only with DBD, except for the C18:1 n9c sample. Notably, there is no statistically significant difference between the samples of C and P_80:5_+PC for C17:1. Across all four samples, C18:1 n9c has the highest value, while C17:1 has the lowest value. In terms of total MUFAs, P_80:5_+PC, C, PC-P, and P_80:5_−PC rank from highest to lowest, respectively.

#### PUFA

The addition of PC, DBD treatment, and their combined effect had varied impacts on the omega-3 and omega-6 groups. Generally, DBD treatment led to a decrease in total omega-6 values and an increase in total omega-3 values compared to the C sample. However, the combined effect of PC and DBD treatment increased the total amounts of both omega-6 and omega-3 compared to the C sample. Across all four samples, C18:3 n3 and C18:2 n6c exhibited the highest amounts, while C17:1 had the lowest amount. It’s crucial to note that if the value of n3/n6 is less than 0.2, it can be harmful to humans. This value increased to 1.17 in the sample treated with DBD compared to the C sample. Conversely, the combined treatment of DBD and PC increased this value to 0.22, suggesting a more balanced omega-3 to omega-6 ratio.

### Changes of color in *Oncorhynchus mykiss rainbow* fillets treated with DBD and PC during 18 days at 4 °C

#### a*

There is a notable increase in the a* values, representing the redness/greenness, of all samples treated with PC, with and without DBD treatment, over the 18-day storage period (p < 0.05) (Supplementary Table 9). The C sample exhibits the greatest increase in a* values during storage, rising from 4.80 to 12.55 (p < 0.05). Despite all treated samples having significantly higher a* values than the C sample on the first day, by the end of the 18th day, the a* values of all treated samples are significantly lower than that of the C sample (*p* < 0.05). Additionally, increasing the voltage and time in DBD-treated samples results in an increase in the a* values (*p* < 0.05). On the 18th day, the sample with the lowest a* value is P_80:5_−PC, which is not significantly different from samples P_70:2_−PC, P_70:5_−PC, P_80:2_−PC, P_80:2_+PC, and P_80:5_+PC. Conversely, the C sample has the highest a* value on the 18th day.

#### b*

The values of b*, representing the yellow/blue color component, were measured regularly over the 18-day storage period (Supplementary Table 10). Results indicate a significant increase in b* value across all samples during storage, with the lowest value observed for the P_70:2_−PC sample (16/11) and the highest for the P_80:5_−PC sample (22/36) on day 18. The C sample exhibited the highest rate of increase in b* value (5.64 to 18.87), followed by the PC-P sample (22.90 to 34.18). Additionally, b* values were significantly higher for DBD-treated samples, both with and without PC, compared to the C sample. Moreover, the b* value increased with increasing voltage and time for all samples throughout the storage period. These findings collectively suggest that DBD treatment and PC contribute to enhancing the color stability of yellowtail fillets during storage.

#### L*

The results indicate a significant decrease in L* values for all samples until the 18th day of storage (*p* < 0.05) (Supplementary Table 11). Initially, DBD treatment alone led to a notable reduction in L* values compared to the C sample until the sixth day. However, by the end of the 18th day, samples treated with P_80:2_−PC, P_70:2_−PC, and P_70:5_−PC showed higher L* values than the C sample. Throughout the 18-day storage period, the rate of reduction in L* values were faster for C samples, P_80:5_−PC, and P_80:5_+PC compared to other samples. Nevertheless, by the 18th day, there was no statistically significant difference observed among these three samples (*p* > 0.05). Notably, the reduction rate of L* values in P_70:2_+PC and P_70:5_+PC samples was slower than in other samples over the 18 days, and by the 18th day, no significant statistical difference was observed between these two samples (*p* > 0.05). On the first day, pretreatment with PC, with or without DBD treatment, resulted in a decrease in L* value with a significant difference compared to the C sample. However, by the end of the 18th day, the L* value of samples pretreated with PC, with or without DBD treatment, was significantly higher than that of the C sample (72.57). Furthermore, the study investigated the effects of increasing voltage and treatment time on L* values. On the third, ninth, and twelfth days, no significant statistical difference was observed between P_70:2_+PC and P_80:2+_PC samples when increasing the voltage from 70 to 80 kV at a constant time of 2 min. However, in all cases, increasing the voltage from 70 to 80 kV at a constant time of 5 min resulted in a significant decrease in L* values. On the 6th, 9th, and 18th days, increasing the treatment time from 2 to 5 min at a constant voltage of 70 kV showed no significant statistical difference in L* values for P_70:2_+PC and P_70:5_+PC samples. However, at a constant voltage of 80 kV, increasing the time from 2 to 5 min led to a significant decrease in L* values for these two samples.

#### ΔE

Throughout the 18-day storage period, all samples display an increase in ΔE values, signifying browning (Supplementary Table 12). The C sample registers the highest ΔE value on the 18th day compared to the first day, while the P_80:5_+PC sample exhibits the lowest increase. Additionally, samples treated solely with DBD show higher ΔE values than those treated with both DBD and PC. Notably, the incorporation of PC reduces the ΔE value in the P_80:5+_PC sample from 12.29 to 8.14.

#### OES

The intensity of species and spectral lines of nitrogen molecules, oxygen atoms, and OH molecule in DBD plasma at 80 kV (Supplementary Fig. 3), with a focus on the wavelength range of 300–400 nm showing prominent peaks corresponding to excited nitrogen species. Additionally, a current voltage diagram is provided for voltages of 70 and 80 kV (Supplementary Figs. 4 and 5).

### Changes of antioxidant activity in *Oncorhynchus mykiss rainbow* fillets treated with DBD and PC during 18 days at 4 °C

#### DPPH

The DPPH scavenging capacity of samples treated with DBD alone does not show a significant difference from the C sample until the third day (*p* > 0.05) (Table 5). However, starting from the sixth day, there is a significant decrease (*p* < 0.05) in the DPPH scavenging capacity of certain samples compared to the C, including P_70:5_−PC, P_80:2_−PC, and P_80:5_−PC. On the first day, the PC-P sample exhibits a significant decrease (*p* < 0.05) in DPPH scavenging capacity compared to the C. Throughout the 18-day period, samples treated with both DBD and PC show significantly lower DPPH scavenging capacity compared to the C and samples treated with DBD alone. However, there is no significant difference (*p* > 0.05) in the DPPH scavenging capacity between samples treated with PC with and without DBD treatment over the 18-day period. On the 18th day, the C sample exhibits the highest DPPH value (36.84), while samples treated with PC, with and without DBD treatment, show the lowest DPPH values (5.48). Results suggest that the combination of DBD and PC treatments may have a more pronounced effect on antioxidant activity than either treatment alone.

**Table 5:** DPPH changes with DBD treatment at 70 and 80 kV voltages and durations of 2 and 5 min, with and without PC treatment, on *Oncorhynchus mykiss rainbow* fillets during an 18-day storage period at 4 °C.

DPPH	Day1	Day3	Day6	Day9	Day12	Day15	Day18
C	11.47 ± 0.1622(a)(A)	13.72 ± 0.2732(a)(B)	15.26 ± 0.5355(a)(C)	17.44 ± 0.3250(a)(D)	20.90 ± 0.3693(a)(E)	23.72 ± 0.7620(a)(F)	36.84 ± 0.9695(a)(G)
P70:2−PC	11.03 ± 0.1137(a)(A)	12.77 ± 0.5013(a)(B)	14.16 ± 0.2524(ab)(C)	16.44 ± 0.3475(a)(D)	19.44 ± 0.3761(ab)(E)	18.91 ± 0.0000(b)(F)	29.62 ± 0.6302(b)(G)
P70:5−PC	10.54 ± 0.0521(a)(A)	12.12 ± 0.4233(a)(B)	13.36 ± 0.1728(b)(C)	15.33 ± 0.2016(b)(D)	17.86 ± 0.1495(b)(E)	15.95 ± 0.4853(c)(F)	24.56 ± 0.6463(c)(G)
P80:2−PC	10.54 ± 0.0521(a)(A)	12.12 ± 0.4233(a)(B)	13.23 ± 0.0404(b)(C)	15.33 ± 0.2016(b)(D)	17.53 ± 0.3921(b)(E)	14.96 ± 0.1626(c)(F)	22.52 ± 0.4869(cd)(G)
P80:5−PC	10.54 ± 0.0521(a)(A)	12.12 ± 0.4233(a)(B)	13.10 ± 0.1003(b)(C)	15.33 ± 0.2016(b)(D)	17.25 ± 0.2433(b)(E)	14.57 ± 0.2850(c)(F)	21.61 ± 0.0000(d)(G)
PC-P	4.54 ± 0.4481(b)(A)	4.90 ± 0.4170(b)(B)	5.31 ± 0.4053(c)(C)	5.22 ± 0.1562(c)(D)	5.54 ± 0.6880(c)(E)	5.66 ± 0.2421(d)(F)	6.12 ± 0.2826(e)(G)
P70:2+PC	6.29 ± 0.1505(b)(A)	4.85 ± 0.3607(b)(B)	5.24 ± 0.3403(c)(C)	5.10 ± 0.1213(c)(D)	4.91 ± 0.3981(c)(E)	5.42 ± 0.2240(d)(F)	5.79 ± 0.2667(e)(G)
P70:5+PC	4.46 ± 0.4182(b)(A)	4.78 ± 0.3974(b)(B)	5.15 ± 0.3839(c)(C)	5.01 ± 0.1624(c)(D)	5.29 ± 0.7467(c)(E)	5.25 ± 0.2089(d)(F)	5.66 ± 0.1978(e)(G)
P80:2+PC	4.49 ± 0.4029(b)(A)	4.82 ± 0.3793(b)(B)	4.99 ± 0.4512(c)(C)	5.06 ± 0.1416(c)(D)	5.33 ± 0.7329(c)(E)	5.31 ± 0.2396(d)(F)	5.73 ± 0.2312(e)(G)
P80:5+PC	4.34 ± 0.3493(b)(A)	4.65 ± 0.3229(b)(B)	4.97 ± 0.3142(c)(C)	4.87 ± 0.0845(c)(D)	5.11 ± 0.6463(c)(E)	5.15 ± 0.2258(d)(F)	5.48 ± 0.2569(e)(G )

Lowercase letters indicate significant changes in the column and uppercase letters indicate significant changes in the row.

#### FRAP

FRAP of the PC-P sample significantly surpasses both the C sample and samples treated with DBD alone (*p* < 0.05) throughout the 18-day storage period (Supplementary Table 13). On the 18th day, the P_80:5_+PC sample exhibits the highest FRAP value (20.57), with no significant difference from the P_80:2_+PC sample (*p* > 0.05), while the C sample records the lowest FRAP value (9.26). Furthermore, the C sample experiences the highest increase in FRAP during the 18-day period (16.87–9.26), whereas the PC-P_80:5_ sample shows the lowest increase (32.74–20.57) (*p* < 0.05). These findings suggest a notable enhancement in the antioxidant activity of treated samples with the addition of P_80:5_−PC and PC-P, particularly evidenced by the substantial improvement in the P_80:5_+PC sample. Moreover, the study underscores the potential utility of these compounds as natural preservatives for extending the shelf-life of food products.

#### ABTS

The results indicate that ABTS values are not significantly different in samples treated solely with DBD compared to the C sample on the first day (*p* > 0.05) (Supplementary Table 14). However, from the third day onwards, ABTS values in the P_80:2_−PC and P_80:5_−PC samples decrease significantly compared to the C sample (*p* < 0.05). Furthermore, except for the first day, ABTS values in samples treated with DBD alone increase from 70 to 80 kV, resulting in a significant decrease compared to the C sample (*p* < 0.05). Conversely, samples treated with PC, with and without DBD, exhibit an improvement in ABTS radical scavenging capacity. Specifically, on the first day, ABTS values decreased significantly from 4.47 in the C sample to 1.54 in the PC sample (*p* < 0.05). Moreover, over the 18-day period, there is no statistically significant difference in ABTS values between samples treated with PC with and without DBD treatment (p > 0.05). On the 18th day, the C sample has the highest ABTS value of 12.58, while the P_80:5_+PC sample has the lowest value of 2.52. However, there is no significant statistical difference between the P_80:5_+PC sample and the P_80:2_+PC and P_70:5_+PC samples. Findings suggest that combining PC with DBD treatment could effectively enhance the ABTS radical scavenging capacity in food samples.

### Changes of sensory properties (color, odor, texture, and overall acceptance) in *Oncorhynchus mykiss rainbow* fillets treated with DBD and PC during 18 days at 4 °C

**Figure 3:**
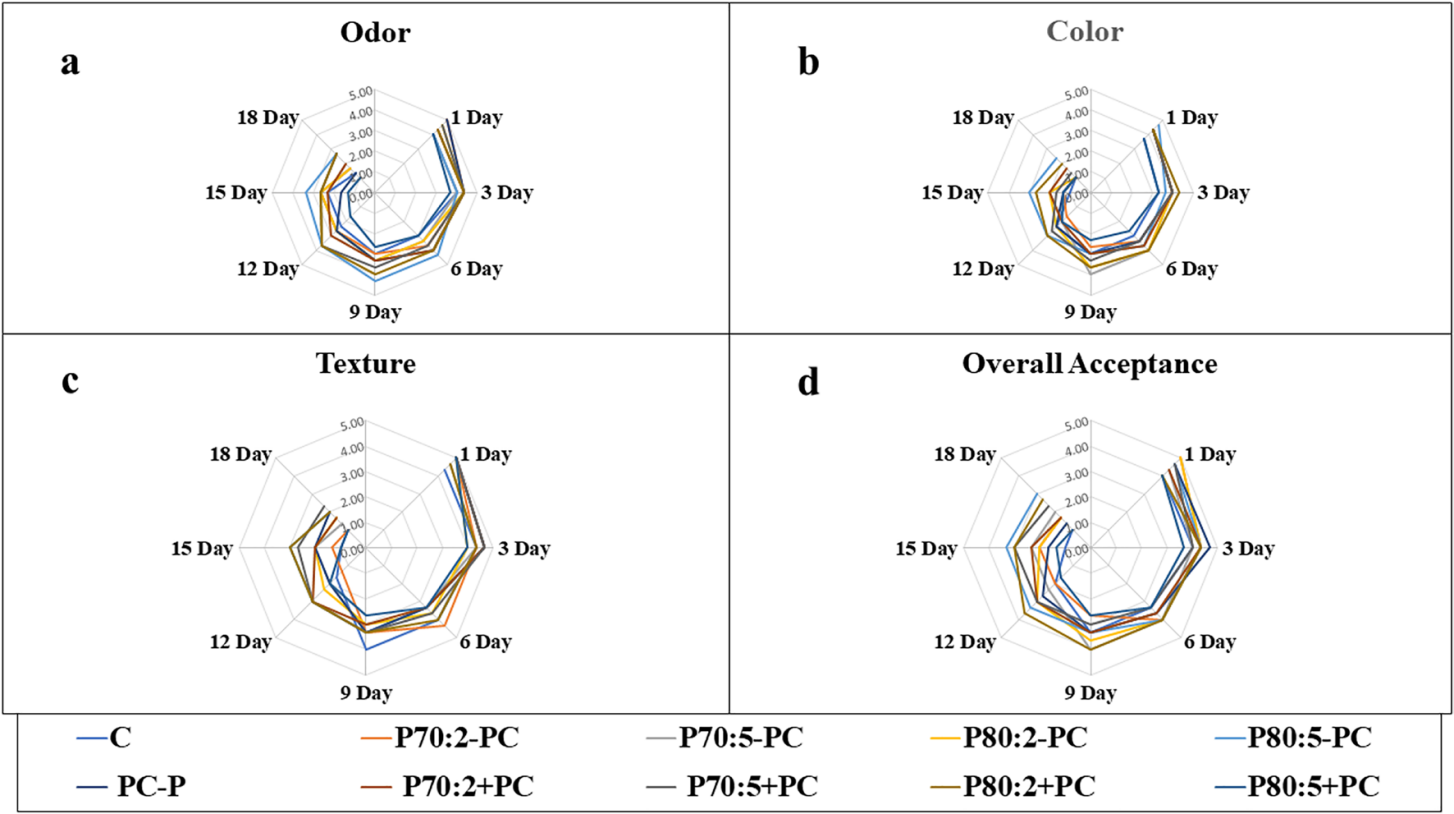
The sensory analysis of *Oncorhynchus mykiss rainbow* fillets over 18 days: (**a**) Odor, (**b**) Color, (**c**) Texture, and (**d**) Overall acceptance.

Results indicate no significant difference in color, odor, texture, and overall acceptability between the C and treated samples (*p* > 0.05) (Fig. 3). However, by the 18th day, the color of P_80:5_−PC and P_80:2_+PC samples exhibited superior conditions compared to the C sample. Additionally, odor conditions of P_80:5_−PC, P_70:5_+PC, and P_80:2_+PC samples surpassed those of the C sample. Tissue quality of P_80:5_−PC, PC-P, P_70:5_+PC, and P_80:2_+PC samples also showed improvement over the C sample on the 18th day. Moreover, both P_80:5_−PC and P_80:2_+PC samples demonstrated better overall acceptance than the C sample.

## Discussion

The study aimed to prolong the shelf life of *Oncorhynchus mykiss rainbow* fillets at 4°C for eighteen days. Two treatment voltages (70 and 80 kV) and two treatment times (2 and 5 min) were examined in DBD. Additionally, the combined effect of DBD with PC at a concentration of 0.065 mg/ml was investigated. In our previous study^14^, we investigated the stability, consistency, and quality of the phycocyanin pigment, confirming its stability at a temperature of 4 °C. Results show that DBD alone extended the fillets’ shelf life compared to the C sample, with the combined DBD and PC treatment demonstrating significantly greater effectiveness than either treatment alone.

Fish contains antioxidants, and employing strong antimicrobial methods like DBD effectively extends its shelf life. Combining DBD with the antioxidant-rich PC helps balance oxidative stress in fish tissue. PC supplements DBD treatment by stabilizing free radicals and minimizing oxidative reactions in fish tissue. Antioxidants like PC donate electrons to free radicals, stabilizing them and reducing their reactivity. Utilizing PC, a plant-based antioxidant, enhances DBD treatment efficacy and extends fish shelf life by inhibiting oxidative stress.

DBD treatment generates reactive oxygen and nitrogen species (RONS), such as ozone, hydroxyl radicals, atomic oxygen, UV radiation, photons, chemical reaction species, and nitric oxide, which penetrate fish fillets, damaging bacterial cell membranes^32,33^. This induces stress responses in bacteria, leading to cellular damage and death. Additionally, DBD disrupts bacterial biofilms^34^, exposing bacteria to antimicrobial agents and environmental stresses^35^. The plasma produced by DBD contains hydrogen peroxide (H2O2), which penetrates bacterial cells, causing oxidative damage and death^36^. DBD effectively decontaminates fish fillet surfaces, reducing initial microbial load and limiting bacterial growth during storage^37^. Increasing treatment time and voltage boosts RONS production, enhancing antimicrobial activity^38^. Longer treatment times allow deeper penetration of RONS, providing extended exposure to bacteria^39^. Higher voltage intensifies plasma reactions, increasing RONS production and antimicrobial efficacy^38^. The combination of prolonged treatment and higher voltage amplifies bacterial inactivation. PC possesses antimicrobial properties, disrupting bacterial membranes and altering pH, hindering bacterial growth. It may limit nutrient availability, further inhibiting bacterial proliferation^40^. Additionally, according to Abou Elmaaty et al.^41^, the presence of phenols in phycocyanin pigment can confer antimicrobial properties to this pigment. The combined treatment synergistically increases antimicrobial activity, damages bacterial cells, and disrupts biofilms, thereby reducing microbial load on fish fillet surfaces, extending shelf life, and maintaining quality. Additionally, PC penetrates bacterial cells more effectively through the pores created by DBD treatment, inducing cell death. Higher voltage and longer treatment times facilitate the penetration of the pigment, resulting in enhanced effects on microbial and chemical parameters. Consequently, the combined treatment exhibits superior outcomes at elevated voltage and duration settings. In this study, increasing the voltage from 70 to 80 kV and extending the duration from 2 to 5 min during DBD treatment effectively reduced the tested bacteria. Combining DBD as a potent oxidative and antimicrobial method with PC as a robust stabilizing and antioxidant agent yielded significantly better results in inhibiting microbial load compared to individual treatments.

Certainly, given that PC can counterbalance ROS, ozone, and free radicals generated by DBD, it can be inferred that UV radiation, photons, charged particles, and other reactive species in DBD, with phenols in PC, play a significant role in inhibiting the microbial load of the samples. In contrast to the findings of Kim et al.^42^, which suggested that DBD had no effect on lactobacillus reduction, it is observed that lactobacillus is reduced by DBD treatment, both with and without PC, compared to the C sample (*p* < 0.05). As reported by Olatunde et al.^8^, Enterobacteriaceae were completely inactivated in DBD-treated samples with treatment durations longer than five minutes. This finding confirms our results regarding the correlation between increased treatment duration and the reduction of Enterobacteriaceae after 18 days of storage.

Initially, the pH slightly increases post-treatment but declines over the eighteen-day period, with the control sample exhibiting the highest pH and P_80:5_+PC sample the lowest. Basic compound accumulation during nucleotide and protein breakdown elevates fish fillet pH, linked to protein denaturation and acidic group reduction^23^. Subsequent days witness pH decreases possibly due to H + ion breakdown and nitric/nitrogen acid production^30^. DBD plasma generates reactive oxygen and nitrogen species (RONS), potentially altering salmon flesh pH via interactions with tissue components and plasma species reacting with water^43^. Increasing DBD voltage and treatment time enhances nonaqueous phase liquid (NAPs) formation due to intensified plasma generation and tissue interaction, allowing deeper reactive species penetration. However, during storage, declining DBD-generated reactive species reduce NAPs formation. Interactions with tissue compounds and environmental factors like temperature and oxygen exposure further inhibit NAPs stability during storage^44^. PC, acting as an antioxidant, could mitigate DBD-induced oxidative processes, potentially stabilizing pH compared to DBD treatment alone.

Objective indices for lipid oxidation, such as PV and TBARS, are commonly utilized to assess primary and secondary lipid oxidation products, respectively. Pseudomonas species containing lipase and phospholipase enzymes can increase free fatty acid levels, prone to oxidation and leading to unstable lipid hydroperoxide production. Elevated peroxide value often results from endogenous lipase or microbial lipase activity^45^. The decrease in fish fillet peroxide value suggests inhibition of lipid oxidation due to PC and DBD treatment^46,47^. The combined treatment of DBD with PC demonstrates significantly greater potency than individual treatments, evident in the TBARS value of P_80:5_+PC across all days. PC's ability to neutralize ROS generated by DBD contributes to its remarkable protective effect on lipids against oxidative processes.

After eighteen days, TVN and TMA content in each fillet stays below one unit daily. Increasing DBD treatment voltage and duration reduces TVN and TMA compared to the control (*p* < 0.05). DBD with PC shows the lowest TVN and TMA levels (*p* < 0.05), while PC alone reduces values compared to the control on most days (*p* < 0.05). According to Olatunde et al.^15^, reduced microbial load correlates with lower TVN and TMA. DBD and PC's combined antimicrobial properties, especially at higher voltage and time, rapidly reduce bacterial growth and non-protein compound deamination. Longer storage boosts microbial proliferation, elevating TVN due to protein degradation^48^. DBD and PC treatments effectively inhibit microbial growth and control TVN increase from protein breakdown. DBD with PC also reduces H2S-producing bacteria, lessening TMA conversion^23^.

DBD with PC reduces H2S-producing bacteria, affecting TMAO to TMA conversion. MUFA and PUFA, considered beneficial, remain stable, unaffected by DBD, PC, or oxidation processes, confirmed by TBARS and PV analysis. According to Kulawik et al.^49^, The TBARS rise over eighteen days, despite unchanged fatty acid composition, suggests non-lipid molecules contribute to TBARS formation. PC's antioxidant activity delays MUFA and PUFA degradation, sensitive to lipid oxidation^50^. SFAs, including palmitic acid, prevalent in fish, are highest in P_80:5_−PC due to DBD-induced ROS, reduced in P_80:5_+PC by PC's antioxidant effects^23,51,52^. The World Health Organization recommends an n-3/n-6 ratio not less than 0.2 to prevent heart problems. The C sample's ratio falls below this standard. Remarkably, the combination of DBD and PC treatment raises this ratio (0.22). DBD alone elevates it to 0.17, while PC alone decreases the ratio even further than the C (0.13).

Antioxidant activity serves as a vital measure of protein bioactivity. According to Bao et al.^28^, optimizing voltage and treatment time in DBD treatment can enhance antioxidant activity. Consequently, fish fillets treated with DBD show improved antioxidant activities, achieved by increasing the treatment voltage to 80 kV and extending the treatment time to 5 min. Oxidative stress, a contributor to various cell disorders and diseases, can be mitigated by PC, which effectively protects cells against oxidative damage caused by ROS. The high ABTS levels in PC highlight its potential as an effective additive^53^. In comparison to previous studies^53^, the combined effect of PC and DBD treatment significantly reduces DPPH and ABTS levels, with P_80:5_+PC exhibiting the lowest levels across all days. This observation aligns with previous research on DBD and PC treatments^5,54,55^. Notably, ascorbic acid oxidation likely contributes to the notable decrease in DPPH observed in all fillets treated at 80 kV starting on the sixth day of storage compared to the control^56^. Furthermore, the FRAP assay demonstrates a significant enhancement in antioxidant electron-donating capacity with PC and DBD treatment. Overall, the combined treatment of DBD and PC exhibits robust effects on the antioxidant activities of fish fillets, complementing the positive effects of DBD treatment alone.

Color plays a crucial role in the appeal of fish to consumers. Research by Albertos et al.^43^, supports our findings, indicating that at low voltages and increasing treatment time, there is no significant change in color factors, aligning with our observations at 70 kV and treatment duration from 2 to 5 min. Conversely, at high voltages and increasing treatment time, there are changes in color factors, with an increase in a* and b* and a decrease in l*, consistent with our study results^32^. Notably, DBD treatment alone did not alter ΔE, and only in the combined treatment with increasing voltage and time did ΔE show a slight decrease^57^.

Although noticeable color changes were observed on the first day due to the treatments' initial impact on the fish fillets, over the eighteen-day period, the most significant changes were observed in the C sample, followed by the sample treated with PC. Subsequently, samples treated with DBD showed better preservation, with the combined treatment samples exhibiting the least color changes overall, particularly notable at 80 kV and 5 min. Hence, it can be concluded that DBD treatment at low voltages has minimal impact on fillet color, with changes becoming more evident with increased time and voltage, albeit mitigated in combined treatments.

The color analysis from sensory evaluations reveals that treated fillets received higher scores for visual appeal compared to the C, confirming the positive impact of PC on food aesthetics. Moreover, sensory analysis indicates that treatments, with or without PC, had no discernible effect on fish texture, likely due to the brief duration of PC soaking and DBD treatment (2–5 min), aimed at preserving texture integrity while leveraging potential effects. As documented in previous research^53^, PC prevents fish fillet color changes by inhibiting the conversion of Fe_3+/_Ferricyanide to Fe_2+_ and controlling the Fe_2+_ complex. Furthermore, oxidative reactions involving PC and DBD contribute to changes in the a*-axis, with combined treatment showing fewer alterations. Despite the anticipated impact of the natural color of PC on the a* factor, its short treatment duration prevented significant effects on this color factor and texture. The slight increase in TBARS corresponds to a decrease in the a* value, often linked to MDA-induced reduction in a* content in fish meat^32^.

The initial changes in factor b* on the first day may be attributed to experimental conditions and the inherent blue color of PC, which can enhance yellowness as a complementary effect when interacting with fish flesh components. Additionally, the Maillard reaction, a chemical process between amino acids and reducing sugars during heating, contributes to the formation of brown pigments that intensify yellowness in food products^58^. PC, interacting with amino acids in fish fillet, can potentially trigger or augment the Maillard reaction, further enhancing yellowness^59^. While PC is recognized for its antioxidant properties that can preserve food color by inhibiting oxidation, its interaction with other fillet components may influence color development. This interaction may have a greater impact on yellowness than on redness or greenness. Moreover, color-forming molecules like myoglobin and hemoglobin can undergo oxidation reactions due to ROS, leading to darkening of fish flesh^60^. Hydrogen peroxide, a primary ROS component, can react with myoglobin, causing fish yellowing during storage^61^. Furthermore, amino acids and free radicals with carbonyl groups can participate in the Maillard reaction, exacerbating yellowness in fish^62^.

The alterations in the l* factor may result from protein denaturation and texture changes^63^. However, it's noteworthy that the treatments did not adversely affect the l* factor, supporting the findings of texture analysis in sensory evaluations. Changes in ΔE can arise from enzymatic and non-enzymatic reactions, melanin formation, the generation of brown products, and caramelization processes^64^. Factors such as high-water activity, alkalinity, and the presence of iron and copper in fish fillets during storage contribute to the development of the brown color^65^. DBD treatment, through its interaction with the alkaline fish surface, intensifies browning due to the active species it produces. However, despite this effect, the combination of DBD with PC treatment significantly mitigates the adverse effects of DBD on fish browning over an eighteen-day period. The treatment's positive impact on ΔE results from minor changes in a* and L* offsetting significant changes in b*. The synergistic effect of these factors contributes to the overall improvement in the ΔE factor.

Consumers may not accept fish if it has a bad odor and lowers sensory scores. TVN and TBARS values are correlated with sensory scores. During the course of the 18-day storage period, the decrease in sensory scores corresponded with an increase in TBARS and TVN values. Additionally, the rise in these values during the DBD treatment contributed to the samples' lower sensory scores. However, taking into account the TBARS and TVN values, the samples' general acceptance within 18 days was deemed acceptable. Fish with bad taste and smell can also be caused by lipid oxidation, which lowers sensory scores. As can be observed, PC and DBD together significantly reduced TVN and TBARS values as well as lipid oxidation. Accordingly, on day eighteen, the samples treated with DBD plus PC were in better condition than the control sample^16^. However, because of the antioxidant qualities of the PC and the rise in FRAP, the fillets containing PC have better color conditions than the control sample^53^.

On the first day, treatments showed no notable impact on the smell, texture, color, or overall acceptance of fish fillets. These factors are crucial for consumer acceptance, as unpleasant odors can diminish sensory scores. Throughout the 18-day storage period, a decrease in sensory scores corresponded to an increase in TBARS and TVN values, indicating lipid oxidation. However, despite these changes, the overall acceptance of samples remained acceptable based on TBARS and TVN values. By the 18th day, samples treated with DBD and PC together were in better condition than the C sample. Additionally, samples treated with PC displayed improved color conditions compared to the C, attributed to the antioxidant properties of PC and increased FRAP.

## Conclusions

In conclusion, the utilization of PC and DBD plasma presents a promising approach for extending the shelf life of *Oncorhynchus mykiss rainbow* fillets. Our study demonstrates that treatments with PC and DBD plasma effectively reduce lipid oxidation, inhibit microbial growth, and preserve sensory attributes, ultimately enhancing the overall quality and prolonging the shelf life of *Oncorhynchus mykiss rainbow* fillets. The combined treatment exhibits synergistic effects, with PC contributing antioxidant properties and DBD plasma offering antimicrobial benefits. These findings highlight the potential of incorporating natural pigments and innovative plasma technologies in seafood preservation, addressing both quality and safety concerns in the food industry. Further research and optimization of treatment parameters are warranted to fully harness the benefits of these approaches and facilitate their practical application in commercial settings.

### Supplementary Information

Supplementary Information.

## Data Availability

All data generated or analysed during this study are included in this published article (supplementary information file: S1).
