# Supplementary material for: The synergistic effect of dielectric barrier discharge plasma and phycocyanin on shelf life of Oncorhynchus mykiss rainbow fillets
Source: Sci Rep. 2024 Apr 22;14:9174. doi: 10.1038/s41598-024-59904-9 (PMC11035654; doi:10.1038/s41598-024-59904-9)
Supplement: Supplementary file 1 — Supplementary Information. [file 41598_2024_59904_MOESM1_ESM.docx]

**Supplementary Table 1**. TPC changes with DBD treatment at 70 and 80 kV voltages and durations of 2 and 5 minutes, with and without PC treatment, on *Oncorhynchus mykiss rainbow* fillets during an 18-day storage period at 4°C.

| **TPC** | **Day1** | **Day3** | **Day6** | **Day9** | **Day12** | **Day15** | **Day18** |
| --- | --- | --- | --- | --- | --- | --- | --- |
| **C** | 3.38±0.0077(a)(A) | 5.40±0.0088 (a)(B) | 6.35±0.0045(a)(C) | 7.39±0.0026(a)(D) | 8.28±0.0084(a)(E) | 8.48±0.0290(a)(F) | 9.69±0.0312(a)(G) |
| **P70:2-PC** | 3.28±0.0127 (b)(A) | 5.11±0.0146 (b)(B) | 6.21±0.0058(b)(C) | 6.35±0.0052 (b)(D) | 7.39±0.0036(b) (E) | 8.25±0.0123(b)(F) | 9.23±0.0067(b)(G) |
| **P70:5-PC** | 3.18±0.0067 (c)(A) | 4.88±0.0228 (c)(B) | 6.10±0.0030(c)(C) | 6.20±0.0071(c)(D) | 7.24±0.0079(c) (E) | 8.12±0.0086(c)(F) | 9.05±0.0070(c)(G) |
| **P80:2-PC** | 3.01±0.0104 (d)(A) | 4.39±0.0056(d)(B) | 5.97±0.0147(d)(C) | 6.17±0.0119 (cd)(D) | 7.05±0.0080(d) (E) | 7.88±0.0104(d) (F) | 8.69±0.0312(d)(G) |
| **P80:5-PC** | 2.62±0.0219(e)(A) | 4.02±0.0107(e)(B) | 5.54±0.0218(e)(C) | 6.13±0.0103(d)(D) | 6.76±0.0086(e) (E) | 7.38±0.0036(e)(F) | 8.26±0.0076(e)(G) |
| **PC-P** | 3.18±0.0158 (c)(A) | 4.98±0.0144(f)(B) | 5.75±0.0134(f)(C) | 6.33±0.0055(b)(D) | 7.59±0.0128(f) (E) | 8.41±0.0034(a) (F) | 9.28±0.0113(b)(G) |
| **P70:2+PC** | 3.05±0.0089 (d)(A) | 4.60±0.0432(g)(B) | 5.40±0.0015(g)(C) | 6.17±0.0077(cd)(D) | 7.11±0.0088(g) (E) | 7.96±0.0088(d) (F) | 9.10±0.0109(c)(G) |
| **P70:5+PC** | 2.92±0.0189(f)(A) | 4.38±0.0046(dh)(B) | 5.07±0.0117(h)(C) | 5.48±0.0125(e)(D) | 6.97±0.0096(h) (E) | 7.63±0.00272(f) (F) | 8.53±0.0347(f)(G) |
| **P80:2+PC** | 2.77±0.0317 (g)(A) | 4.28±0.0097(h)(B) | 5.04±0.0165 (h)(C) | 5.35±0.0062(f)(D) | 6.10±0.0070(i) (E) | 7.28±0.0114(g) (F) | 8.39±0.0035(g)(G) |
| **P80:5+PC** | 2.53±0.0224(h)(A) | 3.84±0.0162(i)(B) | 4.75±0.0269 (i)(C) | 4.13±0.0129(g)(D) | 5.75±0.0246(j) (E) | 6.66±0.0246(h) (F) | 7.55±0.0249(h)(G) |

Lowercase letters indicate significant changes in the column and uppercase letters indicate significant changes in the row.

**Supplementary Table 2.**  Staph changes with DBD treatment at 70 and 80 kV voltages and durations of 2 and 5 minutes, with and without PC treatment, on *Oncorhynchus mykiss rainbow* fillets during an 18-day storage period at 4°C.

| **Staph** | **Day1** | **Day3** | **Day6** | **Day9** | **Day12** | **Day15** | **Day18** |
| --- | --- | --- | --- | --- | --- | --- | --- |
| **C** | 0.00±0.0000(a)(A) | 3.03±0.0135(a)(B) | 4.04±0.0026(a)(C) | 5.09±0.0084(a)(D) | 7.39±0.0043(a)(E) | 8.38±0.0027(a)(F) | 9.33±0.0030(a)(G) |
| **P70:2-PC** | 0.00±0.0000(a)(A) | 2.83±0.0275(ab)(B) | 4.27±0.0054(b)(C) | 4.94±0.0111(b)(D) | 7.10±0.0080(b)(E) | 8.12±0.0058(a)(F) | 9.03±0.0072(a)(G) |
| **P70:5-PC** | 0.00±0.0000(a)(A) | 1.62±0.8105(b)(B) | 4.08±0.0031(c)(C) | 4.75±0.0808(c)(C) | 6.97±0.0209(c)(D) | 8.31±0.0044(a)(DE) | 9.25±0.0050(a)(E) |
| **P80:2-PC** | 0.00±0.0000(a)(A) | 2.23±0.1230(ab)(B) | 3.91±0.0108(d)(C) | 4.45±0.0378(d)(D) | 6.59±0.0290(d)(E) | 7.93±0.0074(a)(F) | 8.77±0.0105(a)(G) |
| **P80:5-PC** | 0.00±0.0000(a)(A) | 2.23±0.1651(ab)(B) | 3.69±0.0404(e)(C) | 4.23±0.0124(ef)(D) | 6.32±0.0179(e)(E) | 7.38±0.0027(b)(F) | 7.92±0.0120(bc)(G) |
| **PC-P** | 0.00±0.0000(a)(A) | 3.28±0.0079(a)(B) | 4.20±0.0063(b)(C) | 5.11±0.0116(a)(D) | 5.94±0.0072(f)(E) | 7.15±0.0054(b)(F) | 8.00±0.0063(b)(G) |
| **P70:2+PC** | 0.00±0.0000(a)(A) | 3.05±0.0165(a)(B) | 4.01±0.0036(cd)(C) | 4.89±0.0136(bc)(D) | 5.62±0.0270(g)(E) | 7.28±0.3349(b)(F) | 7.69±0.0219(bcd)(F) |
| **P70:5+PC** | 0.00±0.0000(a)(A) | 2.87±0.0185(ab)(B) | 3.51±0.0620(f)(C) | 4.34±0.0096(de)(D) | 5.39±0.0042(h)(E) | 6.39±0.0032(c)(F) | 7.36±0.0049(cde)(G) |
| **P80:2+PC** | 0.00±0.0000(a)(A) | 2.74±0.0182(ab)(B) | 3.37±0.0028(g)(C) | 4.13±0.0070(f)(D) | 5.12±0.0078(i)(E) | 6.07±0.0053(cd)(F) | 7.09±0.0061(e)(G) |
| **P80:5+PC** | 0.00±0.0000(a)(A) | 2.49±0.0505(ab)(B) | 2.81±0.0136(h)(B) | 3.87±0.0136(g)(C) | 4.68±0.0223(j)(D) | 5.82±0.0158(d)(E) | 7.22±0.3649(de)(F) |

Lowercase letters indicate significant changes in the column and uppercase letters indicate significant changes in the row.

**Supplementary Table 3.** Lb changes with DBD treatment at 70 and 80 kV voltages and durations of 2 and 5 minutes, with and without PC treatment, on *Oncorhynchus mykiss rainbow* fillets during an 18-day storage period at 4°C.

| **Lb** | **Day1** | **Day3** | **Day6** | **Day9** | **Day12** | **Day15** | **Day18** |
| --- | --- | --- | --- | --- | --- | --- | --- |
| **C** | 0.22±0.0306(a)(A) | 3.10±0.0084(a)(B) | 5.38±0.0045(a)(C) | 6.36±0.0049(a)(D) | 7.37±0.0058(a)(E) | 8.26±0.0104(a)(F) | 9.39±0.0033(a)(G) |
| **P70:2-PC** | 0.09±0.0443(b)(A) | 2.86±0.0034(b)(B) | 5.26±0.0036(b)(C) | 6.16±0.010 (b)(D) | 7.18±0.0075(bc)(E) | 8.20±0.0110(a)(F) | 9.26±0.0042(b)(G) |
| **P70:5-PC** | 0.00±0.0000(c)(A) | 2.79±0.0129(b) (B) | 5.03±0.0127(c)(C) | 5.91±0.0198(c)(D) | 6.84±0.0129(d) (E) | 7.96±0.0262(b)(F) | 9.08±0.0075(c)(G) |
| **P80:2-PC** | 0.00±0.0000(c)(A) | 2.51±0.0418(c) (B) | 4.85±0.0054(d)(C) | 5.83±0.0165 (d)(D) | 6.76±0.0131(e) (E) | 7.44±0.0229(c)(F) | 8.73±0.0117(d)(G) |
| **P80:5-PC** | 0.00±0.0000(c)(A) | 2.03±0.0652(d) (B) | 4.74±0.0208(e)(C) | 5.39±0.0061(e)(D) | 6.33±0.0053(f) (E) | 7.28±0.0040(d)(F) | 8.11±0.0157(e)(G) |
| **PC-P** | 0.00±0.0000(c)(A) | 3.35±0.0050(e)(B) | 5.29±0.0033(b)(C) | 6.06±0.0077(f)(D) | 7.19±0.0057(b) (E) | 8.05±0.0211(e)(F) | 9.14±0.0055(f)(G) |
| **P70:2+PC** | 0.00±0.0000(c)(A) | 3.29±0.0063(e) (B) | 4.52±0.0306(f)(C) | 5.68±0.0104(g)(D) | 7.14±0.0082(c) (E) | 7.68±0.0130(f)(F) | 8.99±0.0084(g)(G) |
| **P70:5+PC** | 0.00±0.0000(c)(A) | 3.23±0.0065(e) (B) | 4.29±0.0070(g)(C) | 5.37±0.0064(e)(D) | 6.30±0.0057(f) (E) | 7.26±0.0097(d)(F) | 8.36±0.0082(h)(G) |
| **P80:2+PC** | 0.00±0.0000(c)(A) | 3.04±0.0126(a) (B) | 4.15±0.0079(h)(C) | 5.12±0.0109(h)(D) | 6.16±0.0052(g) (E) | 7.05±0.0069(g)(F) | 8.14±0.0170(e)(G) |
| **P80:5+PC** | 0.00±0.0000(c)(A) | 2.89±0.0118(b) (B) | 3.77±0.0065(i)(C) | 4.75±0.0200(i)(D) | 5.87±0.0066(h) (E) | 6.84±0.0117(h)(F) | 7.88±0.0083(i)(G) |

Lowercase letters indicate significant changes in the column and uppercase letters indicate significant changes in the row.

**Supplementary Table 4.** EB changes with DBD treatment at 70 and 80 kV voltages and durations of 2 and 5 minutes, with and without PC treatment, on *Oncorhynchus mykiss rainbow* fillets during an 18-day storage period at 4°C.

| **EB** | **Day1** | **Day3** | **Day6** | **Day9** | **Day12** | **Day15** | **Day18** |
| --- | --- | --- | --- | --- | --- | --- | --- |
| **C** | 0.00±0.0000(a)(A) | 3.32±0.0011(a)(B) | 5.35±0.0039(a)(C) | 6.39±0.0056(a)(D) | 8.13±0.0076(a)(E) | 9.09±0.0128(a)(F) | 8.49±0.0299(a)(G) |
| **P70:2-PC** | 0.00±0.0000(a)(A) | 3.23±0.0045(ae)(B) | 5.32±0.0065(a)(C) | 6.15±0.0122(b)(D) | 7.38±0.0027(b)(E) | 8.39±0.0030(b)(F) | 8.37±0.0032(b)(F) |
| **P70:5-PC** | 0.00±0.0000(a)(A) | 3.08±0.0134(bf)(B) | 5.13±0.0056(b)(C) | 5.92±0.0233(c)(D) | 7.08±0.0086(c)(E) | 7.83±0.0112(c)(F) | 8.19±0.0048(c)(G) |
| **P80:2-PC** | 0.00±0.0000(a)(A) | 2.87±0.0148(cg)(B) | 4.99±0.0114(c)(C) | 5.68±0.0152(d)(D) | 6.94±0.0111(d)(E) | 7.55±0.0281(d)(F) | 7.79±0.0102(d)(G) |
| **P80:5-PC** | 0.00±0.0000(a)(A) | 2.40±0.0594(d)(B) | 4.92±0.0045(d)(C) | 5.39±0.0036(eg)(D) | 6.38±0.0036(e)(E) | 7.23±0.1589(e)(F) | 6.85±0.0244(e)(G) |
| **PC-P** | 0.00±0.0000(a)(A) | 3.27±0.0041(a)(B) | 5.04±0.0035(e)(C) | 5.81±0.0304(f)(D) | 7.10±0.0082(c)(E) | 8.18±0.0105(bf)(F) | 9.83±0.0112(f)(G) |
| **P70:2+PC** | 0.00±0.0000(a)(A) | 3.14±0.0117(be)(B) | 4.87±0.0109(f)(C) | 5.47±0.0280(e)(D) | 6.88±0.0127(d)(E) | 7.97±0.0080(cf)(F) | 9.34±0.0048(g)(G) |
| **P70:5+PC** | 0.00±0.0000(a)(A) | 3.01±0.0037(f)(B) | 4.40±0.0035(g)(C) | 5.36±0.0039(g)(D) | 6.36±0.0038(e)(E) | 7.88±0.0083(c)(F) | 9.13±0.0074(h)(G) |
| **P80:2+PC** | 0.00±0.0000(a)(A) | 2.86±0.0122(cg)(B) | 4.21±0.0047(h)(C) | 5.11±0.0099(h)(D) | 6.05±0.0141(f)(E) | 7.16±0.0072(e)(F) | 8.39±0.0030(b)(G) |
| **P80:5+PC** | 0.00±0.0000(a)(A) | 2.60±0.0125(h)(B) | 3.97±0.0119(i)(C) | 4.95±0.0087(i)(D) | 5.39±0.0406(g)(E) | 6.80±0.0160(g)(F) | 8.17±0.0042(c)(G) |

Lowercase letters indicate significant changes in the column and uppercase letters indicate significant changes in the row.

**Supplementary Table 5.** PH changes with DBD treatment at 70 and 80 kV voltages and durations of 2 and 5 minutes, with and without PC treatment, on *Oncorhynchus mykiss rainbow* fillets during an 18-day storage period at 4°C.

| **PH** | **Day1** | **Day3** | **Day6** | **Day9** | **Day12** | **Day15** | **Day18** |
| --- | --- | --- | --- | --- | --- | --- | --- |
| **C** | 6.33±0.0088(a)(A) | 6.63±0.0033 (a)(B) | 7.02±0.0203(a)(C) | 7.18±0.0033(a)(D) | 7.39±0.0088(a)(E) | 7.57±0.0115(a)(F) | 7.73±0.0176(a)(G) |
| **P70:2-PC** | 6.35±0.0115(a)(A) | 6.57±0.0033 (b)(B) | 6.90±0.0033(b)(C) | 7.12±0.0088 (b) (D) | 7.30±0.0115(b) (E) | 7.49±0.0088(b)(F) | 7.64±0.0058(b) (G) |
| **P70:5-PC** | 6.41±0.0088(bc)(A) | 6.54±0.0115 (b) (B) | 6.83±0.0088(c)(C) | 7.05±0.0088(c) (D) | 7.19±0.0088(c) (E) | 7.32±0.0067(c)(F) | 7.42±0.0058(c) (G) |
| **P80:2-PC** | 6.33±0.0067(a)(A) | 6.50±0.0058(c) (B) | 6.84±0.0173(c)(C) | 7.09±0.0067(bc) (D) | 7.26±0.0120(b) (E) | 7.38±0.0088(d) (F) | 7.44±0.0058(c) (G) |
| **P80:5-PC** | 6.40±0.0088(b)(A) | 6/45±0.0133 (d) (B) | 6.70±0.0058(d)(C) | 6.95±0.0058(d) (D) | 7.11±0.0058(d) (E) | 7.24±0.0058(e)(F) | 7.37±0.0058(d) (G) |
| **PC-P** | 6.44±0.0115(bcd)(A) | 6.55±0.0058(b)(B) | 6.75±0.0088 (d) (C) | 6.94±0.0058(d) (D) | 7.07±0.0088(e)(E) | 7.28±0.0058(ce) (F) | 7.33±0.0088 (d) (G) |
| **P70:2+PC** | 6.43±0.0088(bcd)(A) | 6.54±0.0058(b) (B) | 6.64±0.0033(e) (C) | 6.87±0.0058(e) (D) | 7.04±0.0033 (e) (E) | 7.17±0.0088(f) (F) | 7.24±0.0058(e) (G) |
| **P70:5+PC** | 6.47±0.0058(d)(A) | 6.49±0.0033(c) (A) | 6.63±0.0058(e) (B) | 6.82±0.0088(f) (C) | 6.97±0.0033(f) (D) | 7.05±0.0088(g) (E) | 7.12±0.0033 (f) (F) |
| **P80:2+PC** | 6.45±0.0058(cd)(A) | 6.48±0.0088(c) (A) | 6.60±0.0100(e) (B) | 6.79±0.0120(f) (C) | 6.89±0.0088 (g) (D) | 7.03±0.0120(g) (E) | 7.18±0.0088 (g) (F) |
| **P80:5+PC** | 6.45±0.0067(bcd)(A) | 6.44±0.0033(d) (A) | 6.54±0.0033(f) (B) | 6.67±0.0088 (g) (C) | 6.75±0.0058(h) (D) | 6.86±0.0058(h) (E) | 7.05±0.0153(h)(F) |

Lowercase letters indicate significant changes in the column and uppercase letters indicate significant changes in the row.

**Supplementary Table 6.** Acidity changes with DBD treatment at 70 and 80 kV voltages and durations of 2 and 5 minutes, with and without PC treatment, on *Oncorhynchus mykiss rainbow* fillets during an 18-day storage period at 4°C.

| **Acidity** | **Day1** | **Day3** | **Day6** | **Day9** | **Day12** | **Day15** | **Day18** |
| --- | --- | --- | --- | --- | --- | --- | --- |
| **C** | 0.13±0.0000(ab)(A) | 0.16±0.0007(a)(B) | 0.18±0.0013(a)(C) | 0.19±0.0015(a)(D) | 0.20±0.0005(a)(E) | 0.22±0.0005(a)(F) | 0.24±0.0013(a)(G) |
| **P70:2-PC** | 0.13±0.0007(ab) (A) | 0.15±0.0013(b)(B) | 0.17±0.0013 (b) (C) | 0.18±0.0000(b) (D) | 0.19±0.0003(b) (E) | 0.21±0.0003(a)(F) | 0.23±0.0005(b) (G) |
| **P70:5-PC** | 0.12±0.0013(ab) (A) | 0.13±0.0006(c) (B) | 0.15±0.0010(c) (C) | 0.17±0.0007(cg) (D) | 0.18±0.0007(c) (E) | 0.20±0.0007(b)(F) | 0.22±0.0007 (c) (G) |
| **P80:2-PC** | 0.12±0.0018(a) (A) | 0.14±0.0010(df) (B) | 0.16±0.00000(de) (C) | 0.17±0.0010(df)(D) | 0.19±0.0015(d) (E) | 0.21±0.0014(c) (F) | 0.22±0.0007(d) (G) |
| **P80:5-PC** | 0.13±0.0006(ab) (A) | 0.14±0.0007(e) (B) | 0.16±0.0010(df)(C) | 0.16±0.0007(eh)(D) | 0.17±0.0010(e) (E) | 0.20±0.0010(b)(F) | 0.21±0.0007(e) (G) |
| **PC-P** | 0.13±0.0000(ab) (A) | 0.15±0.0005(f)(B) | 0.16±0.0007(e) (C) | 0.17±0.0005(bf) (D) | 0.18±0.0010(d) (E) | 0.19±0.0013(b) (F) | 0.20±0.0013(e) (G) |
| **P70:2+PC** | 0.13±0.0007(b) (A) | 0.14±0.0005(dg) (B) | 0.16±0.0007(f) (C) | 0.17±0.0007(dg) (D) | 0.18±0.0005(c) (E) | 0.19±0.0003(d) (F) | 0.20±0.0003(f) (G) |
| **P70:5+PC** | 0.13±0.0010(ab) (A) | 0.14±0.0000(eg) (B) | 0.15±0.0007(c) (C) | 0.16±0.0000(e) (D) | 0.17±0.0007(e) (E) | 0.18±0.0010(e) (F) | 0.19±0.0000(g) (G) |
| **P80:2+PC** | 0.13±0.0000(ab) (A) | 0.14±0.0007(d) (B) | 0.15±0.0000(cf) (C) | 0.17±0.0007(ch) (D) | 0.17±0.0005(e) (E) | 0.18±0.0009(f) (F) | 0.19±0.0009(h) (G) |
| **P80:5+PC** | 0.13±0.0003(ab) (A) | 0.13±0.0010(ce) (B) | 0.14± 0.0010(g) (C) | 0.16±0.0007(i) (D) | 0.17±0.0006(f) (E) | 0.17±0.0007(g) (F) | 0.19±0.0010(g)(G) |

Lowercase letters indicate significant changes in the column and uppercase letters indicate significant changes in the row.

**Supplementary Table 7.** PV changes with DBD treatment at 70 and 80 kV voltages and durations of 2 and 5 minutes, with and without PC treatment, on *Oncorhynchus mykiss rainbow* fillets during an 18-day storage period at 4°C.

| **PV** | **Day1** | **Day3** | **Day6** | **Day9** | **Day12** | **Day15** | **Day18** |
| --- | --- | --- | --- | --- | --- | --- | --- |
| **C** | 0.22±0.0014 (a)(A) | 0.27±0.0022(a)(B) | 0.32±0.0030(a)(C) | 0.36±0.0022(a)(D) | 0.41±0.0016(a)(E) | 0.46±0.0038(a)(F) | 0.55±0.0028(a)(G) |
| **P70:2-PC** | 0.21±0.0008 (a) (A) | 0.27±0.0014(a)(B) | 0.31±0.0025(a) (C) | 0.35±0.0028(b) (D) | 0.38±0.0028(b) (E) | 0.44±0.0022(b)(F) | 0.52±0.0008(b) (G) |
| **P70:5-PC** | 0.21±0.0014 (ab) (A) | 0.25±0.0008(b) (B) | 0.29±0.0022(b) (C) | 0.33±0.0022(c) (D) | 0.37±0.0008(c) (E) | 0.42±0.0022(c)(F) | 0.50±0.0008(c) (G) |
| **P80:2-PC** | 0.20±0.0016 (ab) (A) | 0.24±0.0014(c) (B) | 0.27±0.00030(c) (C) | 0.31±0.0028(df) (D) | 0.35±0.0022(d) (E) | 0.41±0.0014(d) (F) | 0.48±0.0016(d) (G) |
| **P80:5-PC** | 0.20±0.0022 (ab) (A) | 0.22±0.0028(d) (B) | 0.26±0.0028(d)(C) | 0.29±0.0028(e) (D) | 0.34±0.0014(e) (E) | 0.39±0.0014(e)(F) | 0.45±0.0014(e) (G) |
| **PC-P** | 0.21±0.0022 (ab) (A) | 0.23±0.0014(c)(B) | 0.27±0.0016(c) (C) | 0.32±0.0008(cd) (D) | 0.37±0.0014(c) (E) | 0.42±0.0016(c) (F) | 0.48±0.0014(d) (G) |
| **P70:2+PC** | 0.20±0.0014 (bc) (A) | 0.22±0.0008(de) (B) | 0.26±0.0014(cd) (C) | 0.30±0.0014(f) (D) | 0.35±0.0014(d) (E) | 0.40±0.0014(d) (F) | 0.45±0.0014(e) (G) |
| **P70:5+PC** | 0.19±0.0014 (cd) (A) | 0.21±0.0008(ef) (B) | 0.25±0.0022(e) (C) | 0.29±0.0008(e) (D) | 0.34±0.0014(e) (E) | 0.38±0.0008(e) (F) | 0.42±0.0008(f) (G) |
| **P80:2+PC** | 0.18±0.0044 (d) (A) | 0.21±0.0014(f) (B) | 0.24±0.0016(e) (C) | 0.27±0.0014(g) (D) | 0.32±0.0014(f) (E) | 0.36±0.0008(f) (F) | 0.40±0.0022(g) (G) |
| **P80:5+PC** | 0.18±0.0030 (d) (A) | 0.20±0.0008(g) (B) | 0.22±0.0022(f) (C) | 0.26±0.0022(h) (D) | 0.30±0.0008(g) (E) | 0.34±0.0008(g) (F) | 0.38±0.0025(h)(G ) |

Lowercase letters indicate significant changes in the column and uppercase letters indicate significant changes in the row.

**Supplementary Table 8.** TMA changes with DBD treatment at 70 and 80 kV voltages and durations of 2 and 5 minutes, with and without PC treatment, on *Oncorhynchus mykiss rainbow* fillets during an 18-day storage period at 4°C.

| **TMA** | **Day1** | **Day3** | **Day6** | **Day9** | **Day12** | **Day15** | **Day18** |
| --- | --- | --- | --- | --- | --- | --- | --- |
| **C** | 0.12±0.0003(abd)(A) | 0.19±0.0008(a)(A) | 0.23±0.0005(a)(A) | 0.25±0.0008(a)(B) | 0.28±0.0010(a)(C) | 0.31±0.0028(a)(D) | 0.36±0.0003(a)(D) |
| **P70:2-PC** | 0.12±0.0017(abc) (A) | 0.17±0.0005(b)(AB) | 0.22±0.0016(a) (A) | 0.24±0.0005(b) (BC) | 0.27±0.0003(b) (CD) | 0.31±0.0018(a)(D) | 0.34±0.0003(b) (E) |
| **P70:5-PC** | 0.12±0.0006(abc) (AB) | 0.17±0.0006(c) (AB) | 0.21±0.0014(b) (A) | 0.24±0.0005(b) (BC) | 0.27±0.0006(bc) (CD) | 0.30±0.0008(b)(D) | 0.33±0.0003(c) (E) |
| **P80:2-PC** | 0.12±0.0015(abcd) (AB) | 0.16±0.0008(d) (A) | 0.21±0.0015(c) (A) | 0.23±0.0005(c) (BC) | 0.26±0.0037(d) (BC) | 0.27±0.0006(c) (CD) | 0.30±0.0020(df) (D) |
| **P80:5-PC** | 0.12±0.0011(c) (AB) | 0.15±0.0005(e) (A) | 0.19±0.0003(d)(A) | 0.21±0.0011(d) (BC) | 0.22±0.0017(e) (BC) | 0.25±0.0008(d)(CD) | 0.27±0.0014(e) (D) |
| **PC-P** | 0.12±0.0003(ac) (A) | 0.18±0.0005(f)(B) | 0.21±0.0012(b) (C) | 0.23±0.0029(c) (D) | 0.26±0.0008(cd) (E) | 0.29±0.0012(e) (F) | 0.31±0.0014(d) (G) |
| **P70:2+PC** | 0.12±0.0000(abc) (A) | 0.16±0.0006(d) (B) | 0.19±0.0008(d) (C) | 0.22±0.0012(e) (D) | 0.25±0.0008(f) (E) | 0.27±0.0017(c) (F) | 0.30±0.0008(f) (G) |
| **P70:5+PC** | 0.11±0.0003(bd) (A) | 0.14±0.0005(g) (B) | 0.17±0.0014(e) (C) | 0.20±0.0005(f) (D) | 0.23±0.0000(g) (E) | 0.27±0.0008(f) (F) | 0.29±0.0008(g) (G) |
| **P80:2+PC** | 0.12±0.0006(abcd) (A) | 0.14±0.0003(h) (B) | 0.17±0.0012(f) (C) | 0.19±0.0000(g) (D) | 0.22±0.0008(e) (E) | 0.25±0.0012(d) (F) | 0.27±0.0012(e) (G) |
| **P80:5+PC** | 0.12±0.0005(d) (A) | 0.14±0.0005(h) (B) | 0.15±0.0011(g) (C) | 0.18±0.0015(h) (D) | 0.20±0.0000(h) (E) | 0.23±0.0014(g) (F) | 0.24±0.0017(h)(G) |

Lowercase letters indicate significant changes in the column and uppercase letters indicate significant changes in the row.

**Supplementary Table 9.** a* changes with DBD treatment at 70 and 80 kV voltages and durations of 2 and 5 minutes, with and without PC treatment, on *Oncorhynchus mykiss rainbow* fillets during an 18-day storage period at 4°C.

| **a*** | **Day1** | **Day3** | **Day6** | **Day9** | **Day12** | **Day15** | **Day18** |
| --- | --- | --- | --- | --- | --- | --- | --- |
| **C** | 4.80±0.0260(a)(A) | 4.93±0.0524(a)(A) | 5.63±0.0491(a)(B) | 6.46±0.0384(a)(C) | 8.33±0.1386(a)(D) | 10.68±0.0028(ab)(E) | 12.55±0.1770(a)(F) |
| **P70:2-PC** | 5.23±0.2404(b) (A) | 5.44±0.2454(a)(A) | 6.39±0.1433(b)(B) | 7.03±0.1040(ab)(B) | 8.55±0.1304(ab)(C) | 9.17±0.0018(c)(C) | 10.66±0.1873(bc)(D) |
| **P70:5-PC** | 5.91±0.0176(c) (A) | 6.59±0.0088(c) (B) | 7.20±0.0762(c)(C) | 7.59±0.0601(b)(C) | 9.28±0.2003(bc)(D) | 9.41±0.0008(c)(D) | 10.33±0.0649(b)(E) |
| **P80:2-PC** | 6.01±0.0186(c) (A) | 7.20±0.0981(d) (B) | 7.78±0.0764(cd)(B) | 8.57±0.0731(cd)(C) | 9.93±0.2218(cd)(D) | 10.31±0.0006(a)(D) | 10.87±0.2101(bcd)(E) |
| **P80:5-PC** | 6.76±0.0841(d) (A) | 7.02±0.0929(cd) (AB) | 7.48±0.1819(cd)(B) | 8.31±0.1443(bc)(C) | 10.03±0.1613(cd)(D) | 10.39±0.0008(ab)(DE) | 11.05±0.0410(cd)(E) |
| **PC-P** | 6.64±0.0384(d) (A) | 7.27±0.0273(d)(AB) | 8.00±0.1146(de)(B) | 9.17±0.3427(de)(C) | 10.00±0.1069(cd)(D) | 10.73±0.0012(ab)(D) | 11.75±0.1079(e)(E) |
| **P70:2+PC** | 6.88±0.0473(de) (A) | 8.12±0.0624(e)(B) | 8.69±0.0872(ef)(BC) | 9.51±0.2843(ef)(CD) | 9.80±0.3139(cd)(D) | 10.63±0.0017(ab)(E) | 10.97±0.0862(cd)(E) |
| **P70:5+PC** | 7.26±0.0393(e) (A) | 8.81±0.1027(f) (B) | 9.04±0.3372(f)(BC) | 9.67±0.0100(ef)(CD) | 10.17±0.0296(d)(DE) | 10.47±0.0008(ab)(E) | 10.78±0.0896(bcd)(E) |
| **P80:2+PC** | 8.13±0.0186(f) (A) | 9.46±0.0231(g) (B) | 9.89±0.0231(g)(C) | 10.16±0.0306(fg)(C) | 10.65±0.1997(de)(D) | 11.12±0.0012(bd)(E) | 11.29±0.0384(de)(E) |
| **P80:5+PC** | 8.63±0.0393(g) (A) | 9.44±0.0144(g) (B) | 10.11±0.0346(g)(C) | 10.72±0.0821(g)(D) | 11.16±0.0874(e)(E) | 11.55±0.0014(d)(EF) | 11.70±0.0841(e)(F) |

Lowercase letters indicate significant changes in the column and uppercase letters indicate significant changes in the row.

**Supplementary Table 10.** b* changes with DBD treatment at 70 and 80 kV voltages and durations of 2 and 5 minutes, with and without PC treatment, on *Oncorhynchus mykiss rainbow* fillets during an 18-day storage period at 4°C.

| **b*** | **Day1** | **Day3** | **Day6** | **Day9** | **Day12** | **Day15** | **Day18** |
| --- | --- | --- | --- | --- | --- | --- | --- |
| **C** | 5.64±0.1438(a)(A) | 6.37±0.2317(a)(A) | 7.51±0.1244(a)(B) | 8.15±0.0896(a)(B) | 12.22±0.3312(a)(C) | 13.55±0.2171(a)(D) | 18.87±0.0273(a)(E) |
| **P70:2-PC** | 7.15±0.321(b) (A) | 7.37±0.0696(b)(A) | 8.26±0.0693(b)(B) | 8.32±0.0721(ab)(B) | 12.16±0.0889(a)(C) | 13.87±0.0608(a)(D) | 16.11±0.0088(b)(E) |
| **P70:5-PC** | 8.10±0.0100(c) (A) | 8.90±0.0186(c) (B) | 9.15±0.0726(c)(B) | 10.20±0.0788(cd)(C) | 13.86±0.1255(ab)(D) | 14.86±0.1135(b)(E) | 17.12±0.0273(c)(F) |
| **P80:2-PC** | 8.11±0.0410(c) (A) | 8.40±0.0700(c) (A) | 8.56±0.0088(bc)(A) | 9.31±0.1146(bc)(B) | 13.36±0.1495(ab)(C) | 14.76±0.1519(b)(D) | 16.98±0.0145(c)(E) |
| **P80:5-PC** | 9.28±0.02961(c) (A) | 10.05±0.0361(d) (B) | 10.34±0.0608(d)(B) | 11.22±0.0913(d)(C) | 14.46±0.1450(b)(D) | 15.79±0.0961(c)(E) | 18.11±0.0426(a)(F) |
| **PC-P** | 22.90±0.1015(d) (A) | 23.93±0.0384(e)(AB) | 24.60±0.1707(e)(B) | 25.48±0.4359(e)(B) | 28.07±0.7248(c)(C) | 29.94±0.1586(d)(D) | 34.18±0.1562(d)(E) |
| **P70:2+PC** | 23.81±0.1044(e)(A) | 25.37±0.2166(f)(B) | 27.85±0.2229(f)(C) | 28.35±0.2100(f)(C) | 29.85±0.3477(c)(D) | 30.44±0.1609(d)(D) | 33.39±0.1497(d)(E) |
| **P70:5+PC** | 28.69±0.1682(f) (A) | 29.72±0.1744(g) (B) | 30.43±0.2193(g)(B) | 31.97±0.1868(g)(C) | 33.36±0.1136(d)(D) | 34.64±0.2254(e)(E) | 36.02±0.3227(e)(F) |
| **P80:2+PC** | 30.43±0.0608(g) (A) | 31.22±0.2718(h) (AB) | 32.61±0.0794(h)(BC) | 33.26±0.3635(h)(CD) | 34.41±0.6538(d)(DE) | 34.98±0.1833(ef)(EF) | 36.20±0.3371(e)(F) |
| **P80:5+PC** | 31.37±0.2254(h) (A) | 31.68±0.2128(h) (A) | 32.61±0.1873(h)(B) | 33.47±0.0529(h)(C) | 34.33±0.1637(d)(D) | 35.62±0.1044(f)(E) | 36.22±0.1114(e)(E) |

Lowercase letters indicate significant changes in the column and uppercase letters indicate significant changes in the row.

**Supplementary Table 11.** L* changes with DBD treatment at 70 and 80 kV voltages and durations of 2 and 5 minutes, with and without PC treatment, on *Oncorhynchus mykiss rainbow* fillets during an 18-day storage period at 4°C.

| **L*** | **Day1** | **Day3** | **Day6** | **Day9** | **Day12** | **Day15** | **Day18** |
| --- | --- | --- | --- | --- | --- | --- | --- |
| **C** | 69.44±0.1562(a)(A) | 67.15±0.0606(a)(B) | 65.75±0.0100(a)(C) | 63.75±0.0814(ab)(D) | 60.75±0.0462(a)(E) | 59.59±0.2074(a)(F) | 57.72±0.0569(a)(G) |
| **P70:2-PC** | 68.94±0.1068(a)(A) | 65.92±0.0384(b)(B) | 64.66±0.0353(b)(B) | 63.13±0.7169(abc)(C) | 61.11±0.0529(a)(D) | 59.16±0.0458(ab)(E) | 59.59±0.0273(b)(E) |
| **P70:5-PC** | 67.19±0.1122(b)(A) | 65.04±0.0208(cd)(B) | 63.47±0.0317(c)(C) | 62.25±0.0361(cd)(D) | 61.00±0.3035(a)(E) | 59.16±0.0376(ab)(F) | 59.85±0.1186(b)(G) |
| **P80:2-PC** | 66.75±0.0333(cd)(A) | 64.01±0.1017(e)(B) | 62.31±0.0384(d)(C) | 60.69±0.0902(e)(D) | 59.94±0.0700(b)(E) | 59.47±0.1943(a)(F) | 58.99±0.0410(c)(G) |
| **P80:5-PC** | 65.12±0.0888(e)(A) | 62.49±0.1620(f)(B) | 61.75±0.1656(e)(C) | 60.63±0.0939(e)(D) | 59.35±0.1587(b)(E) | 58.22±0.1137(c)(F) | 57.75±0.0088(a)(F) |
| **PC-P** | 67.58±0.0203(b)(A) | 66.07±0.0433(b)(B) | 65.52±0.0608(a)(B) | 64.17±0.2569(a)(C) | 62.82±0.1650(c)(D) | 61.84±0.1120(d)(E) | 59.80±0.1885(b)(F) |
| **P70:2+PC** | 67.17±0.0722(bc)(A) | 65.24±0.0291(c)(B) | 64.18±0.0551(bf)(C) | 63.08±0.0404(abcd)(D) | 62.31±0.0504(c)(E) | 61.43±0.0481(d)(F) | 60.47±0.1822(c)(G) |
| **P70:5+PC** | 66.43±0.0870(d)(A) | 64.71±0.0639(d)(B) | 63.94±0.0722(cf)(C) | 62.65±0.0361(bcd)(D) | 61.20±0.0393(a)(E) | 60.09±0.0260(ae)(F) | 60.63±0.1212(c)(G) |
| **P80:2+PC** | 66.71±0.0462(d)(A) | 65.04±0.0240(cd)(B) | 62.73±0.0353(d)(C) | 61.83±0.0586(de)(D) | 61.28±0.0656(a)(E) | 60.99±0.0751(de)(F) | 59.89±0.0426(b)(G) |
| **P80:5+PC** | 63.83±0.0612(f)(A) | 62.28±0.0481(f)(B) | 61.26±0.2382(e)(BC) | 60.73±0.1552(e)(C) | 59.40±0.2558(b)(D) | 58.32±0.4821(bc)(DE) | 58.07±0.0557(a)(E) |

Lowercase letters indicate significant changes in the column and uppercase letters indicate significant changes in the row.

**Supplementary Table 12.** ΔE changes with DBD treatment at 70 and 80 kV voltages and durations of 2 and 5 minutes, with and without PC treatment, on *Oncorhynchus mykiss rainbow* fillets during an 18-day storage period at 4°C.

| **ΔE** | **Day1** | **Day3** | **Day6** | **Day9** | **Day12** | **Day15** | **Day18** |
| --- | --- | --- | --- | --- | --- | --- | --- |
| **C** | 2.44±0.3233(ab)(A) | 4.22±0.4938(a)(B) | 4.22±0.1616(a)(C) | 4.64±0.1866(a)(D) | 11.47±0.1866(a)(E) | 13.94±0.1616(a)(F) | 19.30±0.3233(a)(G) |
| **P70:2** | 3.03±0.3233(ab) (A) | 4.58±0.3233(b)(B) | 4.58±0.1616(b) (C) | 2.82±0.3233(b) (D) | 9.87±0.1616(b) (E) | 12.50±0.2469(b)(F) | 14.04±0.1616(b) (G) |
| **P70:5** | 2.40±0.1866(ab) (A) | 4.08±0.3233(bc) (B) | 4.08±0.1616(cd) (C) | 2.95±0.1616(c) (D) | 9.11±0.1866(c) (E) | 11.07±0.3233(c)(F) | 12.44±0.3733(c) (G) |
| **P80:2** | 3.01±0.3233(ab) (A) | 4.81±0.1866(cd) (B) | 4.81±0.2469(ce) (C) | 3.26±0.2469(d) (D) | 9.45±0.2469(c) (E) | 10.76±0.0933(d) (F) | 12.75±0.0000(d) (G) |
| **P80:5** | 2.78±0.1866(ab) (A) | 3.64±0.4938(e) (B) | 3.64±0.1616(f)(C) | 2.74±0.1616(e) (D) | 8.44±0.2469(d) (E) | 10.17±0.1866(e)(F) | 12.29±0.1866(e) (G) |
| **PC** | 1.94±0.3233(b) (A) | 3.03±0.1616(bc)(B) | 3.03±0.1616(b) (C) | 3.91±0.1616(b) (D) | 7.85±0.1616(b) (E) | 9.97±0.1866(f) (F) | 14.63±0.1616(b) (G) |
| **PPC70:2** | 2.78±0.1866(ab) (A) | 5.35±0.2469(de) (B) | 5.35±0.1866(d) (C) | 5.38±0.0933(e) (D) | 8.29±0.1616(d) (E) | 9.54±0.0933(c) (F) | 12.38±0.2469(e) (G) |
| **PPC70:5** | 2.54±0.3733(b) (A) | 3.60±0.0933(de) (B) | 3.60±0.1866(e) (C) | 4.28±0.1616(f) (D) | 7.59±0.1866(e) (E) | 9.28±0.3365(e) (F) | 9.99±0.1866(f) (G) |
| **PPC80:2** | 2.29±0.1866(ab) (A) | 4.87±0.6466(e) (B) | 4.87±0.2333(f) (C) | 3.61±0.1616(g) (D) | 7.22±0.1616(f) (E) | 7.90±0.1616(g) (F) | 9.48±0.1866(g) (G) |
| **PPC80:5** | 1.80±0.1866(a) (A) | 3.24±0.1866(f) (B) | 3.24±0.3365(g) (C) | 3.07±0.1616(h) (D) | 5.92±0.1616(g) (E) | 7.56±0.2469(h) (F) | 8.14±0.4938(h)(G) |

Lowercase letters indicate significant changes in the column and uppercase letters indicate significant changes in the row.

**Supplementary Table 13.** FRAP changes with DBD treatment at 70 and 80 kV voltages and durations of 2 and 5 minutes, with and without PC treatment, on *Oncorhynchus mykiss rainbow* fillets during an 18-day storage period at 4°C.

| **FRAP** | **Day1** | **Day3** | **Day6** | **Day9** | **Day12** | **Day15** | **Day18** |
| --- | --- | --- | --- | --- | --- | --- | --- |
| **C** | 16.87±0.0430(a)(A) | 16.53±0.0744(a)(A) | 16.23±0.0430(a)(AB) | 15.63±0.2686(a)(B) | 14.77±0.2616(a)(C) | 12.70±0.1550(a)(D) | 9.26±0.1137(a)(E) |
| **P70:2-PC** | 17.05±0.0744(a) (A) | 16.57±0.0860(a)(AB) | 16.23±0.1137(a) (AB) | 15.80±0.2394(a) (BC) | 15.15±0.2394(ab) (C) | 13.22±0.2394(ab)(D) | 10.85±0.1290(bc) (E) |
| **P70:5-PC** | 17.39±0.0430(ab) (A) | 17.13±0.1550(b) (AB) | 16.62±0.1550(ab) (B) | 15.93±0.1720(a) (C) | 15.24±0.0744(ab) (D) | 13.22±0.1137(ab)(E) | 10.21±0.0744(ab) (F) |
| **P80:2-PC** | 17.91±0.1720(bc) (A) | 17.39±0.1550(b) (AB) | 16.96±0.0860(bc) (BC) | 16.49±0.2616(ab) (C) | 15.58±0.1550(ab) (D) | 13.69±0.1971(bc) (E) | 10.46±0.1971(b) (F) |
| **P80:5-PC** | 18.55±0.1874(c) (A) | 18.12±0.1550(c) (AB) | 17.48±0.1137(c)(BC) | 17.00±0.1137(b) (C) | 16.06±0.1550(b) (D) | 14.38±0.0860(c)(E) | 11.75±0.2979(c) (F) |
| **PC-P** | 32.31±0.1137(d) (A) | 29.30±0.0744(d)(B) | 27.37±0.1489(d) (C) | 25.43±0.0744(c) (D) | 23.41±0.3101(c) (E) | 21.09±0.2394(d) (F) | 17.13±0.2275(d) (G) |
| **P70:2+PC** | 32.62±0.3101(d) (A) | 29.73±0.1137(d) (B) | 28.10±0.1550(e) (C) | 26.42±0.1137(d) (D) | 24.53±0.1489(d) (E) | 22.03±0.2275(e) (F) | 19.11±0.0744(e) (G) |
| **P70:5+PC** | 32.31±0.0430(d) (A) | 30.85±0.0744(e) (B) | 28.92±0.0744(f) (C) | 27.24±0.0744(de) (D) | 25.43±0.0744(e) (E) | 23.32±0.0430(f) (F) | 18.77±0.3749(e) (G) |
| **P80:2+PC** | 32.66±0.0744(d) (A) | 30.85±0.0744(e) (B) | 29.13±0.1137(fg) (C) | 27.54±0.1550(e) (D) | 25.86±0.1137(ef) (E) | 23.50±0.0744(f) (F) | 19.71±0.1137(ef) (G) |
| **P80:5+PC** | 32.74±0.2394(d) (A) | 31.37±0.0744(e) (B) | 29.69±0.0744(g) (C) | 28.06±0.8602(e) (D) | 26.55±0.0430(f) (E) | 24.40±0.0744(g) (F) | 20.57±0.1550(f)(G) |

Lowercase letters indicate significant changes in the column and uppercase letters indicate significant changes in the row.

**Supplementary Table 14.** ABTS changes with DBD treatment at 70 and 80 kV voltages and durations of 2 and 5 minutes, with and without PC treatment, on *Oncorhynchus mykiss rainbow* fillets during an 18-day storage period at 4°C.

| **ABTS** | **Day1** | **Day3** | **Day6** | **Day9** | **Day12** | **Day15** | **Day18** |
| --- | --- | --- | --- | --- | --- | --- | --- |
| **C** | 4.47±0.0999(a)(A) | 4.81±0.0171(a)(A) | 5.02±0.0377(a)(A) | 5.93±0.0517(a)(B) | 7.64±0.2252(a)(C) | 11.92±0.2828(a)(D) | 12.58±0.1186(a)(D) |
| **P70:2-PC** | 4.34±0.1750(a) (A) | 4.63±0.0624(ab)(AB) | 4.29±0.0206(b) (A) | 5.57±0.0924(a) (BC) | 6.33±0.1733(b) (CD) | 7.17±0.3485(b)(D) | 12.03±0.2806(b) (E) |
| **P70:5-PC** | 4.56±0.1016(a) (AB) | 4.55±0.0805(b) (AB) | 3.88±0.0296(c) (A) | 4.98±0.0000(b) (BC) | 5.60±0.1022(c) (CD) | 5.96±0.1414(c)(D) | 9.41±0.0662(b) (E) |
| **P80:2-PC** | 4.16±0.3702(a) (AB) | 3.35±0.0668(c) (A) | 3.49±0.0136(d) (A) | 4.54±0.1780(c) (BC) | 4.93±0.16.90(cd) (BC) | 5.32±0.1621(cd) (CD) | 6.16±0.0374(c) (D) |
| **P80:5-PC** | 4.03±0.4215(a) (AB) | 3.13±0.0284(c) (A) | 3.25±0.0521(e)(A) | 4.29±0.1304(c) (BC) | 4.40±0.2369(d) (BC) | 5.04±0.1221(d)(CD) | 5.48±0.0296(d) (D) |
| **PC-P** | 1.54±0.0264(b) (A) | 1.96±0.0335(d)(B) | 2.08±0.0500(f) (B) | 2.21±0.0505(d) (B) | 2.55±0.0196(e) (C) | 3.03±0.1154(e) (D) | 3.31±0.0321(e) (E) |
| **P70:2+PC** | 1.54±0.0255(b) (A) | 1.88±0.0068(d) (B) | 2.03±0.0516(f) (B) | 24.83±0.0368(d) (C) | 2.44±0.0131(e) (C) | 2.86±0.0666(e) (D) | 3.02±0.0179(e) (D) |
| **P70:5+PC** | 1.54±0.0179(b) (A) | 1.90±0.0301(d) (B) | 2.00±0.0461(f) (BC) | 2.09±0.0448(d) (BC) | 2.27±0.0733(e) (CD) | 2.60±0.1165(e) (DE) | 2.78±0.1162(ef) (E) |
| **P80:2+PC** | 1.54±0.0213(b) (A) | 1.86±0.0129(d) (B) | 1.97±0.0450(f) (BC) | 2.04±0.0557(d) (BC) | 2.15±0.0414(e) (C) | 2.46±0.0899(e) (D) | 2.69±0.0948(f) (D) |
| **P80:5+PC** | 1.54±0.0228(b) (A) | 1.84±0.0224(d) (B) | 1.95±0.0437(f) (BC) | 2.00±0.0413(d) (BC) | 2.10±0.0442(e) (CD) | 2.26±0.0247(e) (D) | 2.52±0.0850(f)(E) |

Lowercase letters indicate significant changes in the column and uppercase letters indicate significant changes in the row.


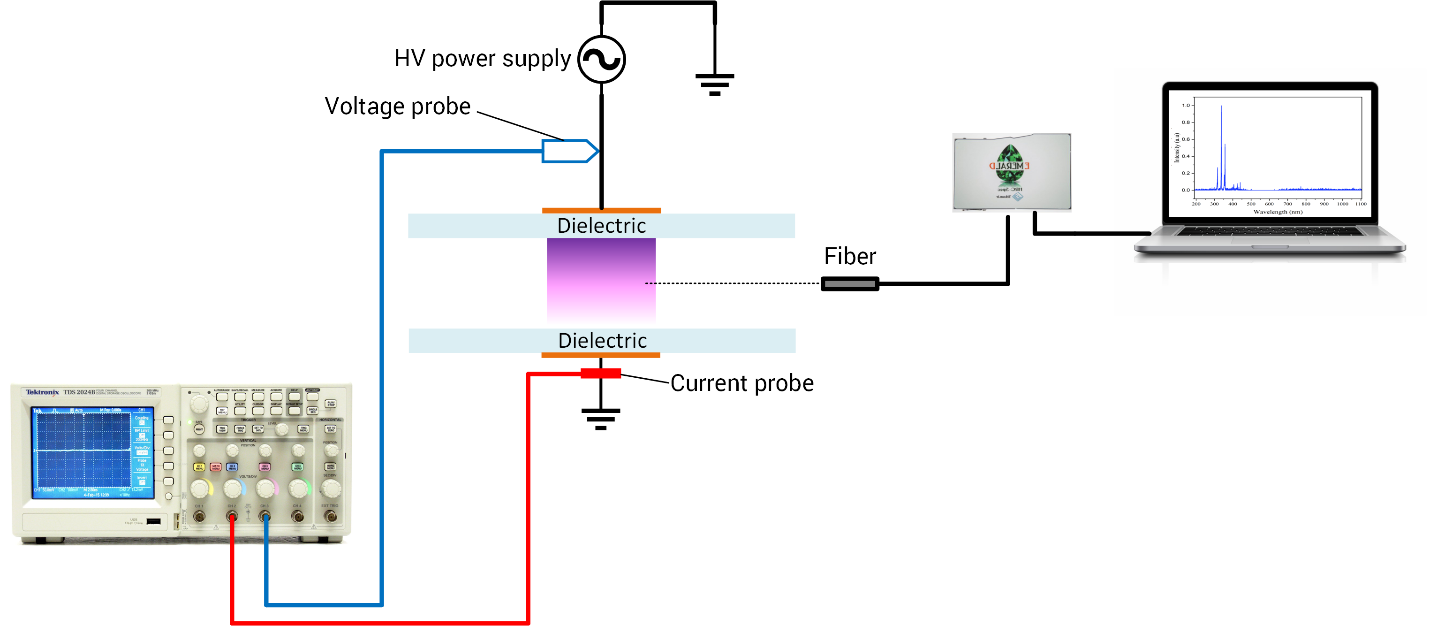


**Supplementary Figure 1**. Schematic of the experimental setup for discharge diagnosis.


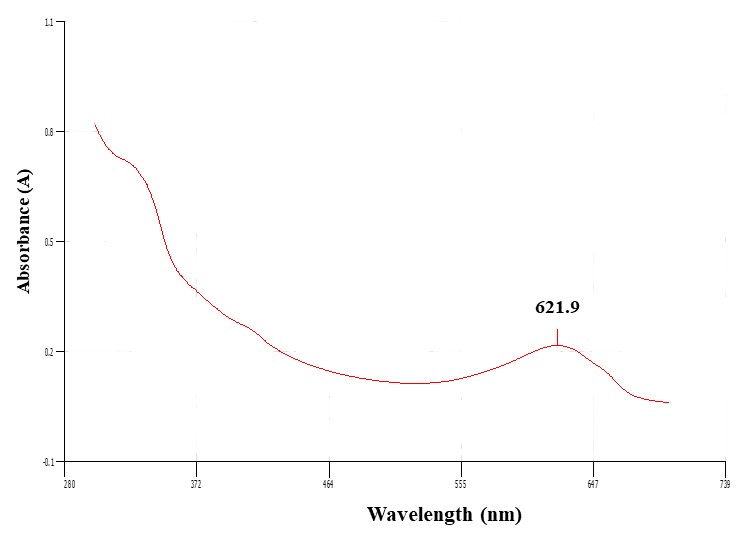


**Supplementary Figure 2.** Purification graph of PC after dialysis


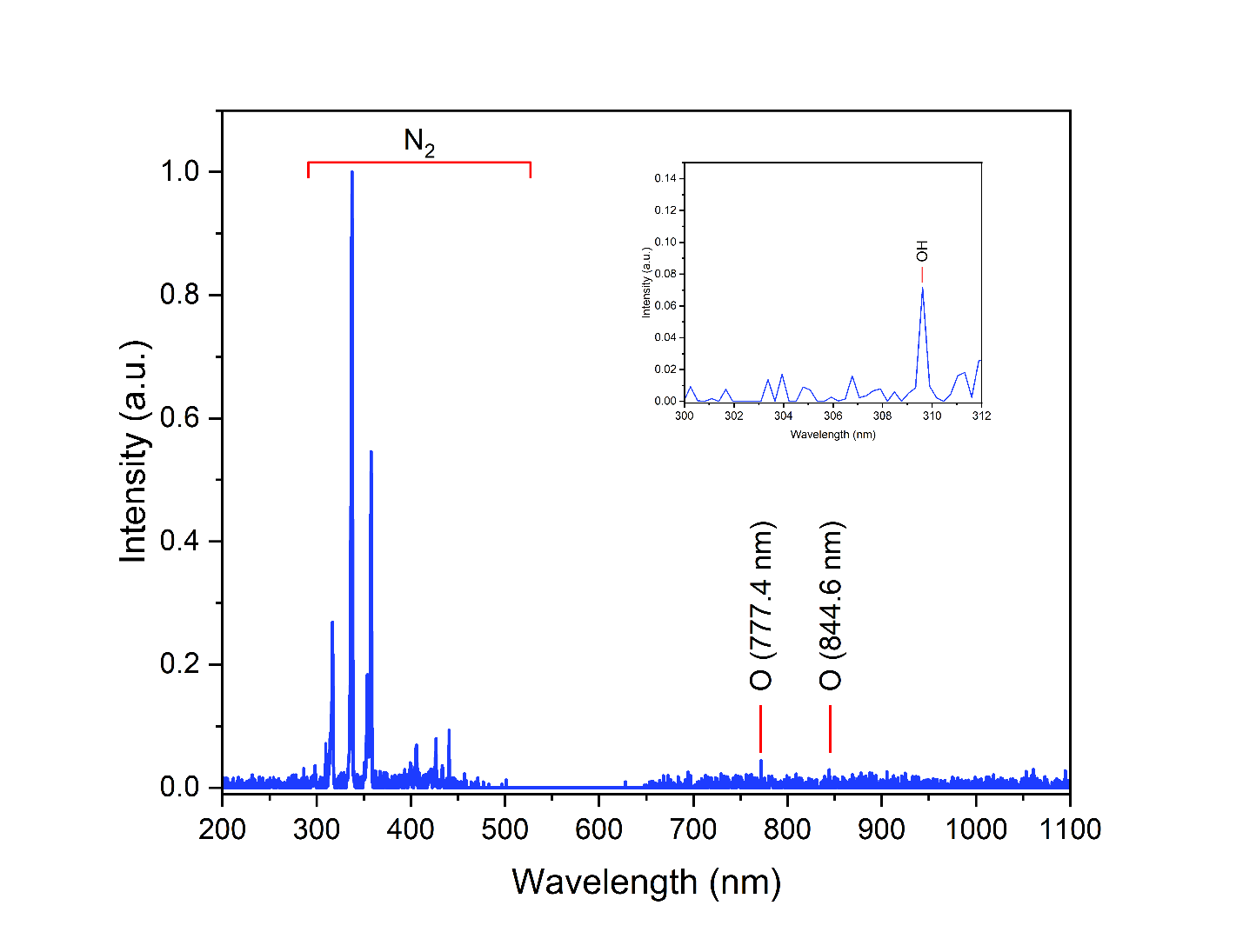


**Supplementary Figure 3.** Density chart of different species produced in DBD.


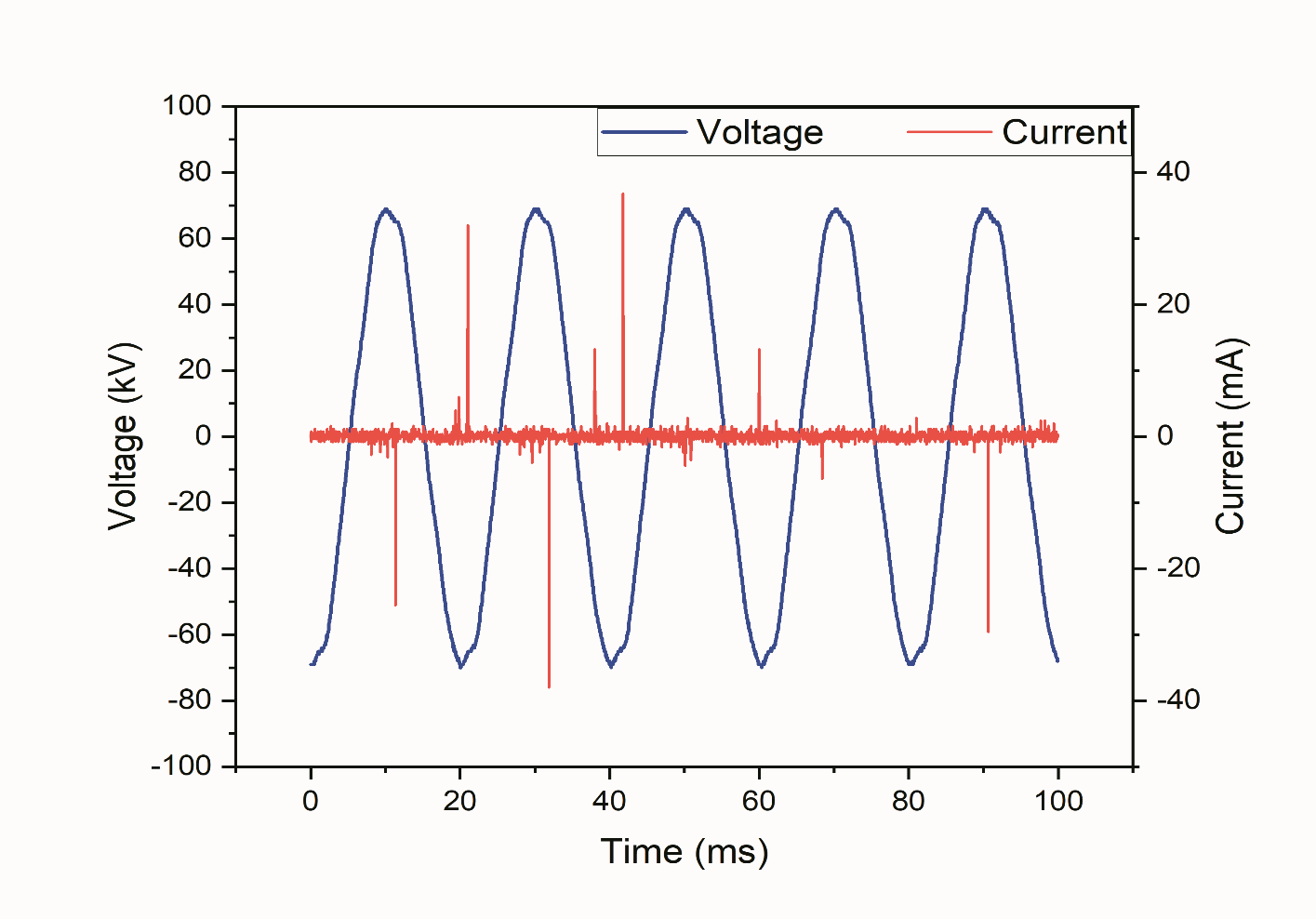


**Supplementary Figure 4.** Voltage and current waveforms of the discharge for voltages of 70


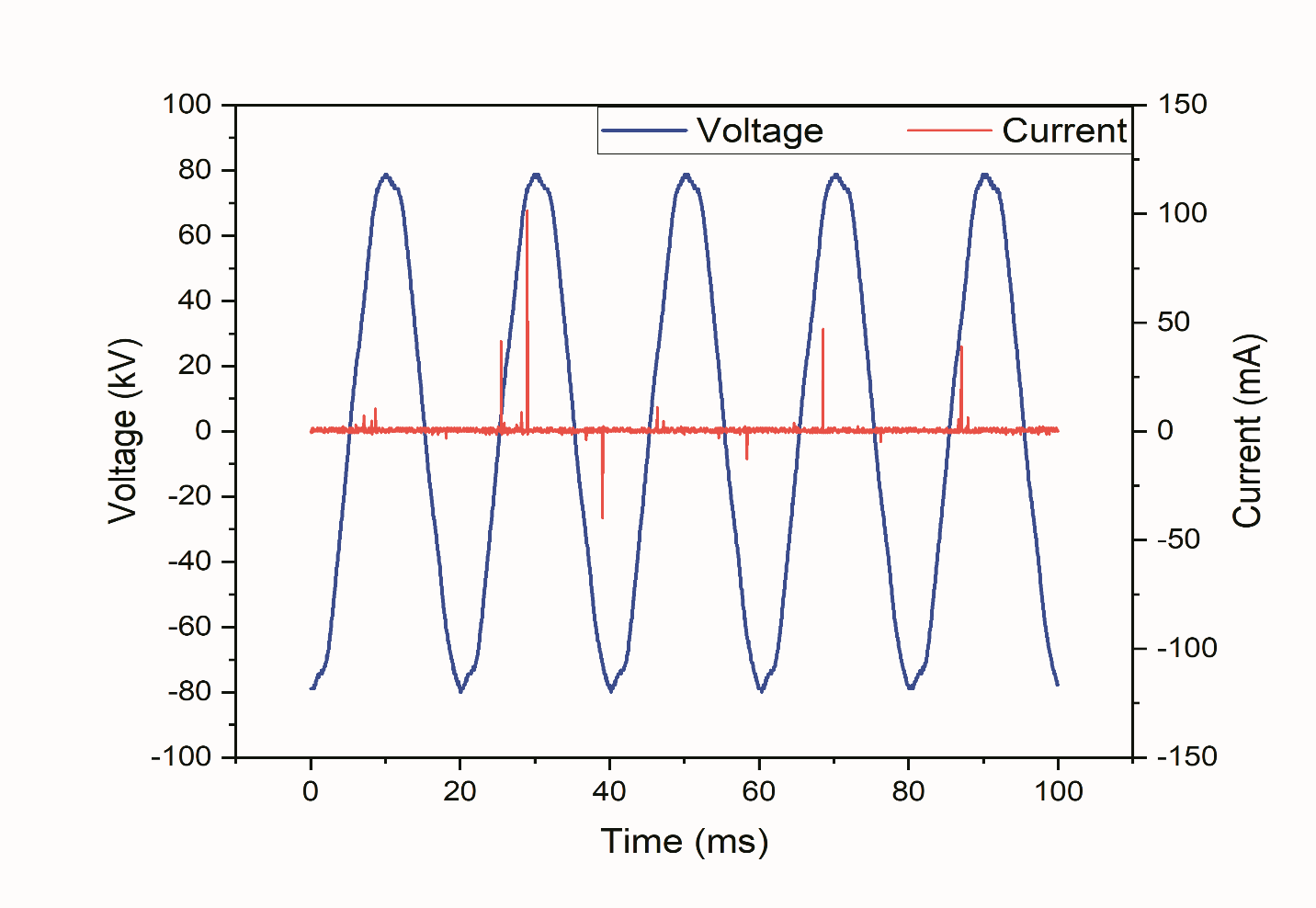


**Supplementary Figure 5.** Voltage and current waveforms of the discharge for voltages of 80
